# Differential deployment of paralogous Wnt genes in the mouse and chick embryo during development

**DOI:** 10.1111/j.1525-142X.2012.00534.x

**Published:** 2012-03

**Authors:** Audrey Martin, Stephanie Maher, Kristen Summerhurst, Duncan Davidson, Paula Murphy

**Affiliations:** a Department of Zoology, School of Natural Sciences, Trinity College DublinIreland; b MRC Human Genetics Unit, Western General HospitalEdinburgh, Scotland

## Abstract

Genes encoding Wnt ligands are crucial in body patterning and are highly conserved among metazoans. Given their conservation at the protein-coding level, it is likely that changes in where and when these genes are active are important in generating evolutionary variations. However, we lack detailed knowledge about how their deployment has diverged. Here, we focus on four Wnt subfamilies (Wnt2, Wnt5, Wnt7, and Wnt8) in mammalian and avian species, consisting of a paralogous gene pair in each, believed to have duplicated in the last common ancestor of vertebrates. We use three-dimensional imaging to capture expression patterns in detail and carry out systematic comparisons. We find evidence of greater divergence between these subgroup paralogues than the respective orthologues, consistent with some level of subfunctionalization/neofunctionalization in the common vertebrate ancestor that has been conserved. However, there were exceptions; in the case of chick Wnt2b, individual sites were shared with both mouse Wnt2 and Wnt2b. We also find greater divergence, between paralogues and orthologues, in some subfamilies (Wnt2 and Wnt8) compared to others (Wnt5 and Wnt7) with the more highly similar expression patterns showing more extensive expression in more structures in the embryo. Wnt8 genes were most restricted and most divergent. Major sites of expression for all subfamilies include CNS, limbs, and facial region, and in general there were more similarities in gene deployment in these territories with divergent patterns featuring more in organs such as heart and gut. A detailed comparison of gene expression patterns in the limb showed similarities in overall combined domains across species with notable differences that may relate to lineage-specific morphogenesis.

## INTRODUCTION

Tight control of cell communication is essential for the organization of any multicellular organism and Wnt signaling pathways constitute one of the major mechanisms utilized by all metazoans. Wnt signaling controls a great variety of cellular events in different contexts including division, differentiation, and migration, and is required for the generation of a normally patterned embryo (Logan and Nusse 2004). Wnt responses are required for establishment of the main body axis early in development of all animals (Lee et al. 2006; Onai et al. 2009; Niehrs 2010) and later in the patterning of multiple systems, for example, the limb (Capdevila and Izpisua Belmonte 2001; Church and Francis-West 2002; Yang 2003; Summerhurst et al. 2008), kidney (Merkel et al. 2007; Yu et al. 2009), and face (Brugmann et al. 2007; Vendrell et al. 2009; Reid et al. 2011). Wnt signaling is also required to maintain adult homeostasis in regenerating adult tissues and inappropriate Wnt signaling is associated with multiple diseases and cancers (Clevers 2006; Klaus and Birchmeier 2008).

This enormous variety of situations in which Wnt signaling operates is matched by complexity in the signaling pathway components and possible cellular responses at every level. The Wnt genes encode secreted glycoproteins of approximately 350 amino acids with conserved Cys residues that are lipid modified (Willert et al. 2003; Takada et al. 2006). They can stimulate both long-range and short-range responses (Zecca et al. 1996; Katanaev et al. 2008) through separate secretion mechanisms (Bartscherer and Boutros 2008). There are 19 Wnt ligand encoding genes in the human and mouse that can signal through a variety of receptors and coreceptors (Frizzled, Lrp, Ror, and Ryk), all encoded by multigene families. Until recently, Wnts were viewed as stimulating one of at least three alternative pathways, however cross-talk between numerous ligands, receptors, coreceptors, and regulators, as well as downstream intracellular messengers has led to a more integrated view of a Wnt signaling network with the outcome depending on the molecular signature and recent history of the system (Kestler and Kuhl 2008; van Amerongen and Nusse 2009; Nalesso et al. 2011). The idea that a single Wnt ligand can activate multiple cellular responses depending on its concentration and the molecular context of the responding cell indicates a system that is highly regulated and finely tuned.

The Wnt ligand is a metazoan invention (Srivastava et al. 2010), and the evolutionary conservation of Wnt pathway components in all metazoan animals underlines the importance of this fundamental network (Lee et al. 2006; Adamska et al. 2010). Wnts are found in all nonbilaterian groups with three Wnt genes in the sponge *Amphimedon* (Adamska et al. 2010) and four in the ctenophore *Mnemiopsis leidyi* (Pang et al. 2010). Surprisingly, the Cnidarian *Nematostella vectensis* genome contains 13 genes grouped in 12 subfamilies (Kusserow et al. 2005; Lee et al. 2006; Cho et al. 2010) all represented in bilaterians. Therefore, expansion of the gene family occurred before Cnidarians and bilaterians diverged. Similarly, it appears that the deuterostome–protostome ancestor possessed 13 subfamilies, 12 of which are still represented in the human genome, although some divergent bilaterian lineages reveal dynamic evolution with duplication, loss, and modification (Cho et al. 2010). The genomes of the common model organisms *Drosophila melanogaster* and *Caenorhabditis elegans* have a paucity of Wnt genes (7 and 5) compared with other protostomes with up to 12 subfamilies retained in some (Janssen et al. 2010). Given the appearance and rapid expansion of Wnt signaling machinery at the time of emergence of organized body plans, and the striking conservation throughout animals, it is not surprising that fundamental roles of Wnt signaling pathways are also conserved with Wnts regulating axis formation and patterning, gastrulation, and germ layer specification in basal metazoans. Posterior Wnt signaling plays a role in body axis formation in a number of invertebrates including Cnidaria and planaria, in the cephalochordate amphioxus, and in vertebrates (Lee et al. 2006; Nordstrom et al. 2006; Onai et al. 2009; Niehrs 2010).

Gene duplication is a major source of evolutionary novelty in animal genomes. The striking conservation of families of developmental regulatory genes across diverse animal groups led to the view that differential expression of conserved sets of regulatory genes accounts largely for body plan diversification (Carroll 2000). The duplication (or loss) of preexisting regulatory genes would therefore be key in diversification; duplications allowing the resulting paralogues to change in their regulatory and/or coding sequences leading to altered morphology (Ohno 1970; Li et al. 2005). Genome analysis indicates that multiple whole-genome duplications (WGD) occurred during vertebrate evolution, in particular two rounds of duplication (2R WGD) at the base of vertebrates (McLysaght et al. 2002; Kasahara 2007; Putnam et al. 2008; Kuraku et al. 2009; Huminiecki and Heldin 2010) with additional duplications in some lineages. The evolution of Wnt genes across all metazoans indicates expansion by duplication in the first animals with an organized body plan followed by a high level of retention in all lineages, with gene loss featuring in some derived species. Vertebrates retain 12 of the 13 subfamilies of Wnt genes but show multiple copies of several, with seven pairs of paralogous Wnt genes in mammals (Janssen et al. 2010) and multiple paralogues in zebrafish (Garriock et al. 2007). A systematic analysis of gene retention following 2R WGD inferred that genes associated with increasing organismal complexity, including morphogenesis, were preferentially retained whereas genes associated with basal cellular functions tended to be excluded (Huminiecki and Heldin 2010). The Wnt gene family was among the most highly expanded.

Ohno proposed that expression divergence is the first step in the divergence of duplicate genes (Ohno 1970). Gene duplicates can be retained if they diverge to acquire new functions (neofunctionalization), including being expressed in new territories, or undergo complementary changes in *cis*-regulatory regions so that both copies are required to carry out all the functions of the ancestral gene (subfunctionalization). Both types of change likely contribute to duplicate retention and permit diversification; comparison of yeast and human data suggests that a large proportion of duplicate genes undergo rapid subfunctionalization followed by a prolonged period of neofunctionalization (He and Zhang 2006). From studies on *Drosophila*, Gu et al. (2004) concluded that duplicated genes increase gene expression diversity both within and between genomes. Studies on the human and mouse genomes revealed increased expression divergence spatially in lineage-specific duplications with paralogous genes tending to become more specialized in their expression patterns, showing decreased breadth and increased specificity as the size of the gene families increased (Huminiecki and Wolfe 2004). Although these arguments suggest that paralogous genes may enable tissue specialization and morphological evolution, little is known about how Wnt paralogues have diverged and how this might contribute to the evolution of vertebrate body plans. Previous studies include comparison of regulatory regions of mouse Wnt5 paralogues, showing conservation of a regulatory module (Kapasa et al. 2010), and comparison of expression of Wnt9 paralogues in zebrafish (Cox et al. 2010).

Chick and mouse lineages diverged approximately 310 million years ago (Hedges 2002) and represent the two best amniote model organisms for which we have a comparatively good understanding of developmental events. Analysis of the chick genome has established synteny with the mouse and human genomes and showed a tendency for greater gene loss (Chick Genome Sequencing consortium 2004). Although the evolutionary distance between the species makes comparisons challenging, the chick is a pivotal model for understanding mammalian evolution with the promise of distinguishing mammalian-derived features and innovations. The evolutionary distance will be an advantage in searching for noncoding regulatory elements (Chick Genome Sequencing consortium 2004).

Here, we carry out cross-species (mouse and chick) comparisons of Wnt paralogue/orthologue expression patterns to address how gene deployment in the embryo has changed between duplicated genes in the same species and in divergent vertebrate lineages. To what extent have the genes maintained their respective expression domains over more than 300 million years of evolution or can we see evidence of divergence that might contribute to the divergence in body plans? We chose to compare four specific sets of paralogous genes (Wnt2, Wnt5, Wnt7, and Wnt8) that in the mouse embryo represent examples where both paralogues are extensively expressed (Wnt5 and Wnt7) or show restricted expression (Wnt2 and Wnt8) and where paralogues have similar (Wnt7) or very divergent (Wnt8) expression domains (Summerhurst et al. 2008). Data for the mouse at Theiler stage (TS) 15, TS17, and TS19 were previously digitally recorded in three dimensions (Summerhurst et al. 2008). Here, data were generated for the chick across Hamburger and Hamilton (HH) stages 20–26 for comparison, encompassing similar embryological events. These stage ranges do not capture the earliest expression domains but allow more complex patterns in the various emerging systems to be analyzed. The data sets were systematically compared; exploring how such full, three-dimensional expression data can be integrated across two model systems. Species-specific differences may indicate evolutionarily or functionally important changes in gene regulation and demonstrate how essential genes that control body patterning and morphogenesis have evolved.

## MATERIALS AND METHODS

### Genome analysis and sequence comparisons

**Fig 1:**
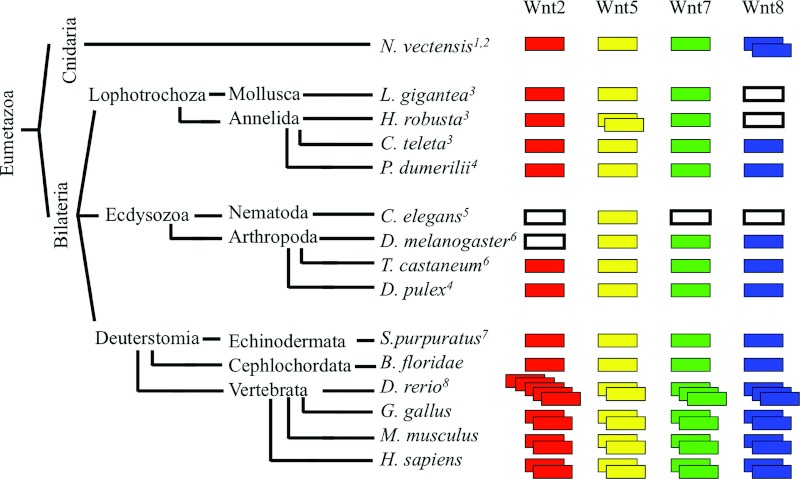
The occurrence of Wnt2, Wnt5, Wnt7, and Wnt8 genes across selected cnidarian and bilaterian species. The number of paralogues is indicated by the number of repeated boxes in each case. Gene loss is indicated by a white box. The phylogenetic relationships are represented by the tree on the left. Compiled from; *^1^*Kusserow et al. 2005; *^2^*Lee et al. 2006; *^3^*Cho et al. 2010; *^4^*Janssen et al. 2010; *^5^*Prud'homme et al. 2002; *^6^*Bolognesi et al. 2008; *^7^*Croce et al. 2006; *^8^*Garriock et al. 2007.

The amphioxus and chick genomes were searched for occurrence of Wnt genes using BLAST tools (http://blast.ncbi.nlm.nih.gov/) (supporting information Table S1). The representation of Wnt2, Wnt5, Wnt7, and Wnt8 genes in additional metazoan lineages was compiled from published literature (Fig. 1). Protein sequences were compared using CLUSTALW (Thompson et al. 2002) and COBALT (http://blast.ncbi.nlm.nih.gov/).

### RNA probes

Details of the probes used to generate the data presented for each of the chick genes is shown in supporting information Table S2. The probes were produced from cDNA inserts in plasmid vectors or from RT-PCR amplified fragments with extended T7 primer sequences attached (David and Wedlich 2001) through in vitro transcription with digoxygenin-labeled RNA nucleotides and T7, T3, or Sp6 polymerase as appropriate (as previously described (Summerhurst et al. 2008).

### Embryo collection

Embryos were collected from fertilized chick eggs (strain: Ross 308 from Enfield Broiler Breeders, Straffan, County Kildare, Ireland) incubated at 37.5°C with 70% humidity for 3–5 days. The embryos were precisely staged using Hamburger and Hamilton criteria (Hamburger and Hamilton 1992) and guidelines presented in Fisher et al. (2008). Stage-matched embryos for all stages between HH20 and HH26 were selected for expression analysis. Embryos were fixed in 4% PFA (Sigma-Aldrich, Ireland), dehydrated in methanol, and stored at –20°C.

### In situ hybridization

In situ hybridization was carried out as described in Summerhurst et al. (2008) except Proteinase k was used at a concentration of 20 μg/ml for 1 min per HH stage of the embryo, posthybridization washes were in 2× SSC (2 × 10 min), 2× SSC/0.1% CHAPS (3 × 20 min), 0.2× SSC/0.1% CHAPS (3 × 20 min) at 65°C, and anti-DIG antibody (Roche, Germany) was used at 1/1000. At least three embryos per stage were hybridized for each probe per experiment and each experiment was carried out at least three times and up to six times. Negative (sense) and positive (a well-characterized gene) controls were used in every experiment. After a suitable level of staining was reached, any trapping in the brain was reduced by washing with PBS. Embryos were viewed superficially and recorded using an Olympus SZX12 microscope with attached camera (Qimaging micropublisher 3.3, Roche, Germany) prior to three-dimensional scanning.

### OPT scanning and three-dimensional reconstruction

Optical projection tomography (OPT) scanning and three-dimensional reconstruction was carried out as described in Summerhurst et al. (2008). Full three-dimensional data are submitted to the Chick Atlas Project for public release.

### Analysis and comparison

AMIRA (Visage Imaging) and Mouse Atlas software were used for visualization and allowed analysis of expression patterns through whole three-dimensional visualizations and by “cutting” virtual sections in multiple planes. The sites of expression were first textually described and tabulated for every stage. A systematic approach was taken to comparing expression patterns between each set of paralogues in the chick, paralogues in the mouse (Summerhurst et al. 2008), and across species orthologues for each of the eight individual genes. The sites of expression for each gene, species, and stage were first textually described and then tabulated (supporting information Tables S3–S10). A framework for comparison was established in which the tabulated descriptions were used to compare across each pair of paralogues and orthologues. Similarities and differences were noted for each territory, combining the stages for summary purposes (supporting information Tables S11–S26), while double-checking the observations against the original three-dimensional data. A scoring system was devised to grade the similarity or difference between two patterns and is represented in supporting information Tables S11–S26; = used to denote where two genes are expressed in largely equivalent domains; * two genes

expressed in the same territory with minor differences in the domains; ** expression in the same territory but in different domains; *** lack of expression in the same territory.

## RESULTS

### The origin and conservation of Wnt paralogues

It is well established that the mouse and human genomes contain the same complement of 19 Wnt genes belonging to 12 subfamilies, with seven paralogous pairs; Wnt2 (2 and 2b), Wnt3 (3 and 3a), Wnt5 (5a and 5b), Wnt7 (7a and 7b), Wnt8 (8a and 8b), Wnt9 (9a and 9b), and Wnt10 (10a and 10b) (e.g., Bolognesi et al. 2008; Janssen et al. 2010). The amphioxus and chick genomes and transcripts were searched for Wnt sequences. Twelve Wnt genes were found in amphioxus representing one member of each of the 12 subfamilies found in mammals, consistent with duplication of the Wnt genes in the vertebrate lineage (supporting information Table S2). Nineteen Wnt sequences were found in the chick genome but not the same complement of subfamily paralogues as in the mouse; only one Wnt10 gene was found and two Wnt11 paralogous genes. The second chick Wnt11 has been previously described and because fish and frogs also possess orthologues of both Wnt11 genes, it represents a vertebrate duplication lost in mammals (Hardy et al. 2008). We conclude that the second Wnt10 paralogue was lost in the avian lineage because the gene was detected in the genome of the anolis lizard but was not found in the genomes or expressed sequence tag (EST) banks of any avian species (chicken, turkey, zebra finch, and mallard). However, the Wnt1 gene, which is physically linked to the Wnt10b gene, is present in the genomes of anolis lizard, zebra finch, and mallard, and in the EST banks of chicken and turkey (D. Burt, personal communication).

Figure 1 illustrates the occurrence of genes from the four subfamilies compared in this work across selected bilaterian and cnidarian species. All four subfamilies are retained in most lineages with loss of Wnt2 in *C. elegans* and *D. melanogaster* and loss of Wnt8 in two lophotochozoan species. *Caenorhabditis elegans* is the most divergent retaining only a Wnt5 orthologue. There are lineage-specific duplications of Wnt8 in *Nematostella* (Lee et al. 2006) and Wnt5 in *Helobdella robusta* (Cho et al. 2010). The same complement of paralogues in these subfamilies is seen in mouse, chick, and human. Zebrafish show extra lineage-specific duplications of Wnt2, Wnt7, and Wnt8 (Garriock et al. 2007).

**Fig 2:**
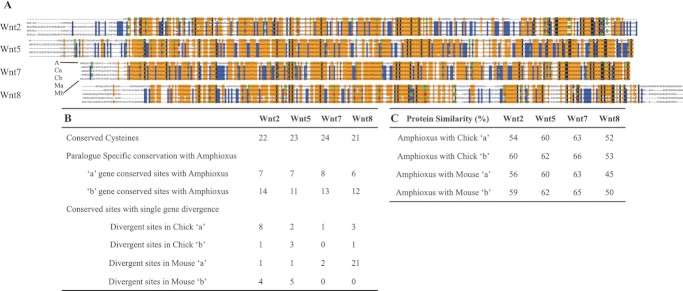
(A) Diagrammatic representation based on the protein sequence alignments of Wnt2, Wnt5, Wnt7, and Wnt8 paralogue proteins from chick and mouse with amphioxus orthologues. This is intended to show the overall pattern of conservation rather than the alignment detail. The order of protein sequences in each case is A, amphioxus; Ca, chick paralogue “a” (2, 5a, 7a, 8a); Cb, chick paralogue “b”; Ma, mouse paralogue “a”; Mb, mouse paralogue “b” as indicated to the left of Wnt7 comparisons. Conserved cystein residues are shown in black. The subgroups are approximately aligned according to the pattern of conserved cysteins. Orange highlighting indicates other residues conserved with amphioxus across at least three vertebrate proteins. Cases of a divergent residue in a single gene are highlighted (emboldened; gap in orange); the number of such cases is tabulated in (B). Blue indicates residues conserved across all four vertebrate proteins but not with amphioxus. Green indicates residues where both “a” paralogues or both “b” paralogues are conserved with amphioxus, in such cases “a” paralogue divergence is shown in purple, “b” paralogue divergence is shown in red (tabulated in B). (B) The number of conserved cysteines; the number of sites showing paralogue-specific conservation with amphioxus (both “a” or “b” paralogues are conserved with amphioxus whereas the other paralogues are not); and the number of sites at which a single subfamily gene is divergent from all other members of the subfamily (mouse, chick, and amphioxus). (C) Percent similarity in all pairwise comparisons with the amphioxus orthologue.

To investigate differential sequence divergence of chick and mouse Wnt subfamily paralogues and orthologues, their protein sequences were aligned with the corresponding amphioxus Wnt proteins (Fig. 2). The overall conservation in each case is striking and the arrangement of conserved cysteines across the subgroups is also noteworthy. Of the genes examined, the Wnt5 and Wnt7 subfamilies are the most highly conserved across chordate and vertebrate representatives; they also show 23 of 24 conserved cysteines characteristics of Wnt proteins whereas Wnt2 and Wnt8 subfamilies have 22 and 21, respectively (Fig. 2B). The emphasis here was to explore differential divergence of either the chick or mouse genes and of either set of paralogues (“a” or “b”). Similarity scores show that in each subfamily, the “b” paralogues are slightly more similar to the amphioxus orthologue than “a” paralogues (Fig. 2C). This is more striking looking at the number of individual residues where “a” or “b” paralogues are specifically conserved with amphioxus (Fig. 2B). However, looking at the position of differentially conserved sites through the proteins (Fig. 2A), although there is some clustering (e.g., Wnt2b-conserved sites toward the carboxy terminus), there is no clear differential conservation of regions within the proteins. The amino terminal region is most divergent in all proteins and the region of greatest divergence of vertebrate subfamilies from amphioxus is located toward the carboxy terminal, between two groups of conserved Cys residues. This region is most divergent from amphioxus in Wnt2 and Wnt8 subfamily members but it is notable in Wnt2 that there is conservation between all vertebrate genes (highlighted in blue). It is also noteworthy that in the Wnt8 subfamily, there is a cluster of five “b” paralogue-specific conserved sites on the amino side of this region.

Relative divergence of individual genes is indicated by sites where a single subfamily member in a single species diverges at sites where all other subfamily genes are conserved (Fig. 2B). By far, the most divergent single gene in this respect is mouse Wnt8a with 21 differences distributed across the protein (overall 45% similarity with amphioxus [Fig. 2C]); chick Wnt2 and mouse Wnt5b show eight and five differences, respectively, at otherwise conserved sites.

### Overview of gene expression patterns

Three-dimensional digital records of the expression patterns of the eight chick Wnt genes across stages HH20–HH26 have been entered in the Chick Atlas Project (http://www.echickatlas.org/ecap/home.html) and are summarized for stages HH20, HH23, and HH26 in supporting information Tables S3, S5, S7, and S9 for Wnt2, Wnt5, Wnt7, and Wnt8 genes, respectively. Whole mouse embryo expression patterns at stages TS15, TS17, and TS19 were previously submitted to EMAGE (Summerhurst et al. 2008) and are described in supporting information Tables S4, S6, S8, and S10.

**Fig 3:**
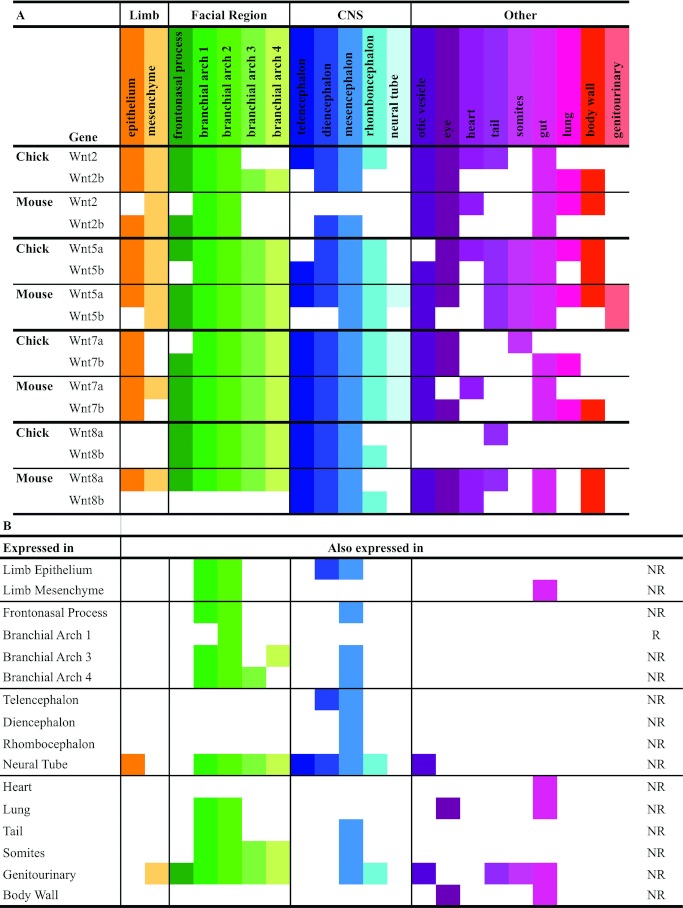
Comparison of gene deployment in anatomical territories of the embryo. (A) Summary of embryonic territories comparing expression of paralogous genes within, and orthologus genes across, mouse and chick species for Wnt subfamilies Wnt2, Wnt5, Wnt7, and Wnt8. This summarizes the data described in supporting information Tables S3–S10. (B) Expression territories shared by all Wnt genes analyzed. In each case, all genes expressed in the territory named on the left are also expressed in the territories indicated in that row. The column on the right indicates if the pattern of shared territories is reciprocal (R) or not reciprocal (NR)—that is, if the territories are also shared in the other direction, for example, all genes expressed in branchial arch 1 are not also shared by the limb epithelium (NR).

Figure 3A summarizes detection of expression in different territories within the embryo; a site is indicated here even if expression is detected at a single stage of development so it represents the similarities and differences in spatial regulation of the expression patterns but does not capture temporal dynamism of the patterns (this is represented in the supporting information Tables S3–S10). Each of the genes we examined showed expression in a variety of body regions with the chick Wnt8 subfamily showing the most restricted patterns. The matrix of genes and structures shown in Fig. 3 displays two striking features. First, each gene we examined has a pattern of expression that is unique both within and between species. Second, most structures express more than one subfamily of Wnt genes, though some express only one or two of the subfamilies we examined (genitourinary system, somites). In particular, with the exception of the branchial arches, each of the structure expresses a unique set of Wnt genes. Branchial arches 1 and 2 share expression of the same genes, as do branchial arches 3 and 4, although it is important to note that analysis at this level does not reveal or compare precise domains of expression within each territory; this is explored below.

Figure 3B indicates expression territories shared by the Wnt genes analyzed with the intention of indicating shared regulation. For example, genes expressed in the limb ectoderm are also expressed in branchial arches 1 and 2, diencephalons, and mesencephalon; genes expressed in the lung are also expressed in branchial arches 1 and 2, eye, and gut. One interesting observation is that expression in any territory of the CNS is shared with expression in the mesencephalon. In addition, all genes expressed in limb epithelium, frontonasal process, branchial arches 3 and 4, the tail, somites, or the genitourinary system are also expressed in the mesencephalon. Another observation is that if a gene is expressed in branchial arch 4, it is also expressed in the more anterior branchial arches. Figure 3B also notes if the commonality is reciprocal or not (right-hand column). The majority of shared territories are not reciprocal, with the exception of branchial arches 1 and 2 and branchial arches 3 and 4.

### Wnt2 gene expression: comparison of orthologues and paralogues

**Fig 5:**
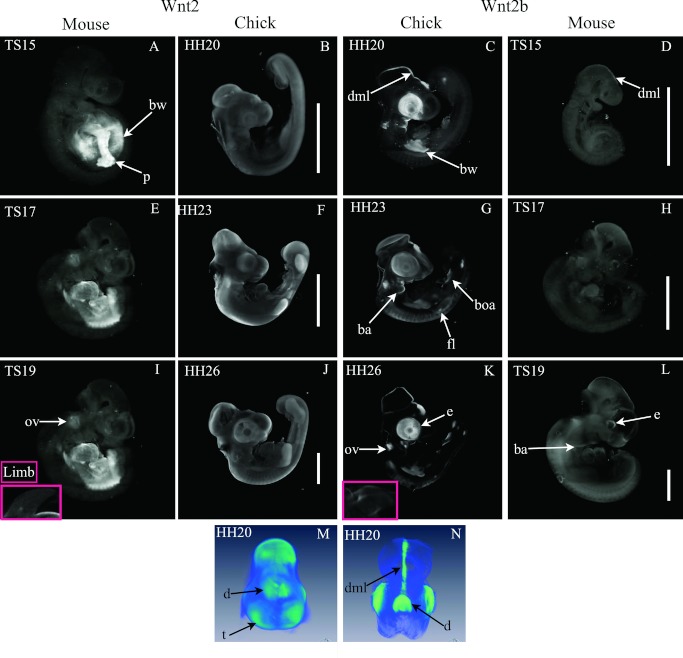
Wnt2 subfamily gene expression in mouse and chick embryos at stages indicated. Main images are external views (volume rendered) of whole embryos following three-dimensional imaging (OPT): mouse Wnt2 (A, E, and I); chick Wnt2 (B, F, J, and M); chick Wnt2b (C, G, K, and N); mouse Wnt2b (D, H, and L). Longitudinal virtual sections through the left forelimb are shown inset in (I and K); (I) is a midline section along the dorso-ventral axis, (K) is posterior of midline in order to show the expression. (M and N) show frontal views of the head highlighting expression (pseudocolored green on-line) in the telencephalon. Scale bars indicate 2 mm and are indicative for each stage. Abbreviations: ba, branchial arches; boa, base of amnion; bw, body wall; dml, dorsal midline; e, eye; fl, forelimb; ov, otic vesicle.

The expression of Wnt2 in the chick embryo was previously described only in the developing eye (Fokina and Forlova 2006), so current descriptions are novel (Fig. 5 and supporting information Table S3). The previously reported chick Wnt2b expression data (Loganathan et al. 2009; Sienknecht and Fekete 2009) are extended here to show localized expression patterns in the CNS and limbs (Fig. 5 and supporting information Table S3). The most prominent expression is in the dorsal midline of the diencephalon and midbrain (Fig. 5N) extending either side of the midline laterally into prosomere 2 in “‘sickle” shaped domains. The paralogous chick gene Wnt2 and the orthologous mouse Wnt2b are also expressed in the dorsal midline but not in this very distinctive “sickle” domain. Mouse Wnt2 expression is not detected in the brain so in this territory chick Wnt2 expression is more similar to mouse Wnt2b, and chick Wnt2b has a distinctive unique domain. In the limb, chick Wnt2b is expressed in a localized domain of mesenchyme and overlying dorsal ectoderm in the proximal limb buds from HH23 (Fig. 5K, inset). Mouse Wnt2b by contrast is expressed in a more extensive domain in the dorsal mesenchyme and ectoderm, which is more similar to limb expression of chick Wnt2; the patch of proximal core mesenchyme expressing mouse Wnt2 is in fact more similar to the expression of chick Wnt2b (Fig. 5I).

**Fig 4:**
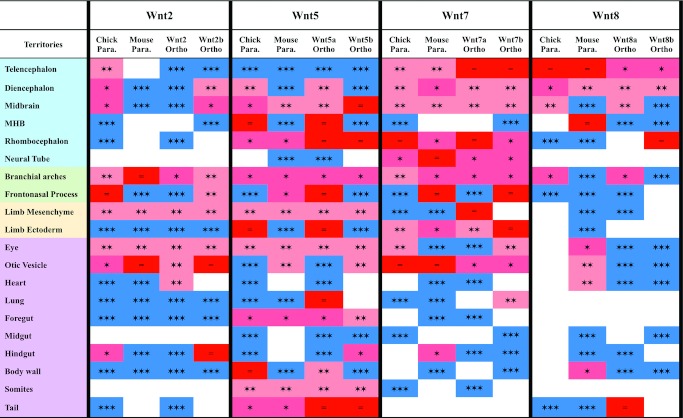
Comparison of territories and domains of embryonic expression of paralogues (within species) and orthologues (across species) for Wnt subfamilies Wnt2, Wnt5, Wnt7, and Wnt8, comparing stages TS15, TS17, and TS19 in the mouse and HH20, HH23, and HH26 in the chick. The headings on the top indicate the genes being compared; for example, chick Wnt2 compares chick Wnt2 and chick Wnt2b; mouse Wnt2 compares mouse Wnt2 and mouse Wnt2b; Wnt2 M versus C compares mouse Wnt2 with chick Wnt2; Wnt2b M versus C compares mouse Wnt2b with chick Wnt2b. Anatomical territories are listed on the left column. Similarities and differences indicated by color code (color available on-line) and symbol are defined as follows: *** that only one of the two genes is expressed in the territory; ** expression in different domains in the same territory; * indicates expression in similar, overlapping but distinct domains; = expression in largely similar domains. The comparisons summarized here are laid out in more detail in supporting information Tables S11–S26)

The Wnt2 genes in the mouse are among the most restricted and are the most divergently expressed paralogues (Fig. 4) with each gene expressed mostly in mutually exclusive territories, for example, Wnt2 in the trunk and Wnt2b in the brain (Fig. 5), with similar expression only in the branchial arches and otic vesicle. Chick Wnt2 genes are expressed in a broader set of tissues, especially noteworthy in the CNS (mouse Wnt2b shows only very restricted expression), limb, and facial region. Wnt2b in the chick is expressed in branchial arches 1 through 4 whereas Wnt2 in both the mouse and chick are restricted to arches 1 and 2; expression through multiple arches is typical of other Wnt genes. Although the chick paralogues are expressed in more common structures/territories, there is very little similarity in the domains, with the exception of the frontonasal process. So while the chick genes are generally more broadly expressed, they also indicate divergent regulation.

The two species utilize Wnt2b very differently in most territories (Fig. 4), with only the hindgut diverticulum and midbrain being similar. Although overall there is very limited similarity in the expression domains of Wnt2 or Wnt2b paralogues in either species or either set of orthologues across the species, there are a number of examples of similarities in expression crossing orthologue groups, as already noted above for the limb: A very prominent region of expression for mouse Wnt2 is in the ventral body wall and placenta (Fig. 5A, E, and I), as previously reported (Monkley et al. 1996; Summerhurst et al. 2008); chick Wnt2 is not expressed in the corresponding territory; however, chick Wnt2b is expressed strongly in the ventral body wall and base of the amnion up to HH23 (Fig. 5C and G). It appears that chick Wnt2b expression shares some aspects of both Wnt2 and Wnt2b in the mouse.

### Wnt5 gene expression: comparison of orthologues and paralogues

**Fig 6:**
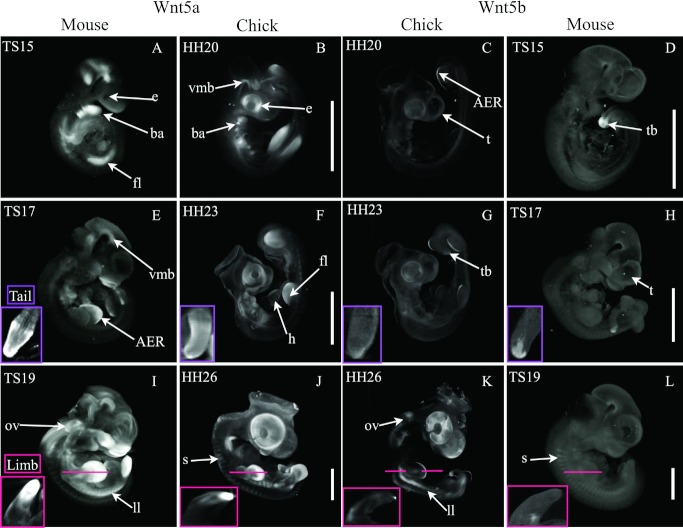
Wnt5 subfamily gene expression in mouse and chick embryos at stages indicated. Main images are external views (volume rendered) of whole embryos following three-dimensional imaging (OPT): mouse Wnt5a (A, E, and I); chick Wnt5a (B, F, and J); chick Wnt5b (C, G, and K); mouse Wnt5b (D, H, and L). Virtual sections through the tail are shown inset in (E, F, G, and H). Longitudinal (dorso-ventral) sections through the limb (plane indicated) are shown inset in (I, J, K, and L). Scale bars indicate 2 mm and are indicative for each stage. Abbreviations: AER, apical ectodermal ridge; ba, branchial arches; e, eye; fl, forelimb; h, heart; ll, lateral line; ov, otic vesicle; s, somites; t, telencephalon; tb, tail bud; vmb, ventral midbrain.

The expression of both Wnt5 paralogues in both species has previously been described extensively (e.g., Loganathan et al. 2005; Summerhurst et al. 2008; Quinlan et al. 2009), especially expression of Wnt5a in the progress zone of the limb bud where we show a gradient with highest levels posteriorly. This work confirms the previously reported sites of expression (Fig. 6 and supporting information Tables S5 and S6) adding three-dimensional digital records of the data and comparative analysis across paralogues and between chick and mouse (Figs. 4 and 6 and supporting information Tables S15–S18).

For Wnt5 genes, most of the anatomical regions we examined show expression of both paralogues in both species. All four genes are expressed in the limb, developing facial regions, central nervous system, and tail bud. Only the mouse expresses Wnt5 genes in the developing genitourinary tract. Overall, Wnt5 genes also show more conservation of expression domains across both paralogues and orthologues than Wnt2 genes (Figs. 4 and 6). The major differences noted in the telencephalon (Fig. 4) reflect the fact that chick Wnt5a and mouse Wnt5b are not expressed whereas the paralogues in both species are; this means the domain is not shared by either set of orthologue or paralogue. This is not a case of a territory occupied by opposite paralogues in different species because the domain of chick Wnt5b and mouse Wnt5a are distinct with chick Wnt5b expressed in anterior forebrain and mouse Wnt5a in the cortical hem. Mouse Wnt5b has more restricted expression in the CNS than other subfamily genes, being expressed only in the ventral midbrain. Both Wnt5 chick paralogues are widely expressed and overlap in most territories. The expression is similar in the apical ectodermal ridge (AER), optic cup, cornea, foregut, body wall, and the tail. Mouse Wnt5 paralogues show fewer similarities, but overlap in the facial region, foregut, somites, and tail. Wnt5a orthologues show a good deal of similarity in brain, facial region, limb, lung, and tail but differ in the posterior gut, heart, otic vesicle, and neural tube. Wnt5b orthologues are similar in the midbrain, hindbrain, branchial arches, and limb mesenchyme but differ in the frontonasal process and limb ectoderm in addition to the forebrain. In general, the chick deploys Wnt5b more extensively than the mouse and there is less conservation of sites across the species compared to Wnt5a (supporting information Tables S17 and S18). In the limb, mouse Wnt5b is not expressed in the AER whereas the chick orthologue is (Fig. 6K and L, inset). There is interesting alternative deployment in a domain within the limb bud with dorsal proximal limb expression detected for mouse Wnt5a and for chick Wnt5b. The posterior tail and tail bud express both paralogues in both species (Fig. E–H, inset). Mouse Wnt5a is more extensively expressed than Wnt5b, particularly in the mesoderm. Chick Wnt5a and Wnt5b are very similarly expressed to their respective mouse orthologues with 5a more extensive than 5b.

### Wnt7 gene expression: comparison of orthologues and paralogues

**Fig 7:**
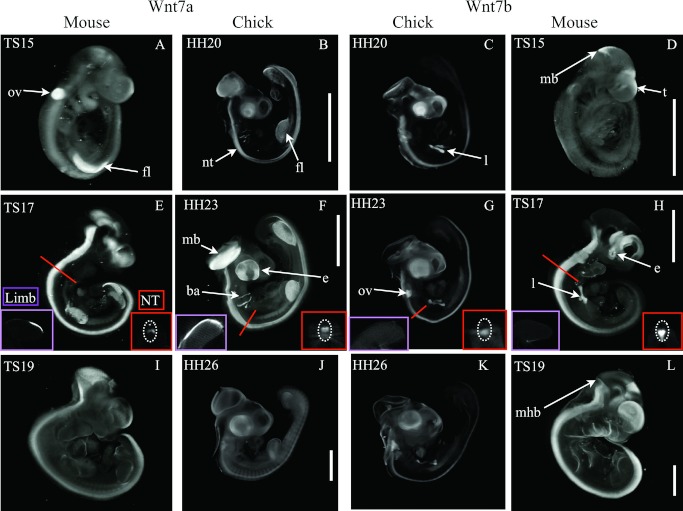
Wnt7 subfamily gene expression in mouse and chick embryos at stages indicated. Main images are external views (volume rendered) of whole embryos following three-dimensional imaging (OPT): mouse Wnt7a (A, E, and I); chick Wnt7a (B, F, and J); chick Wnt7b (C, G, and K); mouse Wnt7b (D, H, and L). Virtual longitudinal sections through the limb (dorsal up, ventral, down) are inset in (E–H) for the respective genes and stages. Virtual transverse sections through the neural tube at the position indicated are also inset in (E–H). Abbreviations: ba, branchial arch; e, eye; fl, forelimb; l, lung; mb, midbrain; mhb, midbrain-hindbrain boundary; nt, neural tube; ov, otic vesicle. Scale bars indicate 2 mm and are indicative for each stage.

Wnt7 is the most highly conserved subfamily across all members (Fig. 2) with conserved sites spread throughout the protein, except the extreme N-terminus. It also shows the highest conservation of expression patterns across paralogues and orthologues (Fig. 4) with most differences seen outside the CNS, facial region, and limbs where all four genes are prominently expressed (Fig. 7).

Wnt7 paralogues in the chick are quite similarly expressed with Wnt7b generally more extensive. Wnt7a has more prominent expression in clearly defined territories, especially in neural, limb, and facial ectoderm. The situation is similar for Wnt7 paralogues in the mouse with Wnt7a more restricted and Wnt7b more extensive (supporting information Tables S7 and S8). Overall, there is also very high conservation of expression domains among orthologues across the species particularly in the CNS, limb, and developing facial structures. This is especially seen where individual paralogues are utilized in specific tissues, for example, Wnt7b in the lungs of both chick and mouse (Fig. 7G and H). In tissues that express both paralogues in both species, paralogue-specific differences are also generally conserved. For example, the neural tube shows expression of both genes in defined territories across the dorsoventral axis with Wnt7a expressed more dorsally than Wnt7b in both species (Fig. E–H, inset). The limb also shows conservation of paralogue-specific domains with Wnt7a expressed in dorsal ectoderm and Wnt7b in proximal ventral ectoderm in both species (Fig. 7E–H, inset). One detailed difference here between species is that at TS19 in the mouse, Wnt7a begins to be expressed also in proximoventral ectoderm as well as distal-dorsal ectoderm (Summerhurst et al. 2008) whereas chick Wnt7a is still restricted dorsally even at HH26. It is possible that Wnt7a is expressed in ventral ectoderm at even later stages. Other differences for Wnt7a include mouse deployment in the gut and heart and chick deployment in the eye. For Wnt7b, the expression is similar in the CNS, facial region, limb, otic vesicle, and lung but there are differences in deployment by the gut, body wall, and midbrain–hindbrain boundary.

As with the Wnt5 subfamily, Wnt7 genes share most of the major sites of expression with differences arising in the spatial extent of the domains.

### Wnt8 gene expression: comparison of orthologues and paralogues

**Fig 8:**
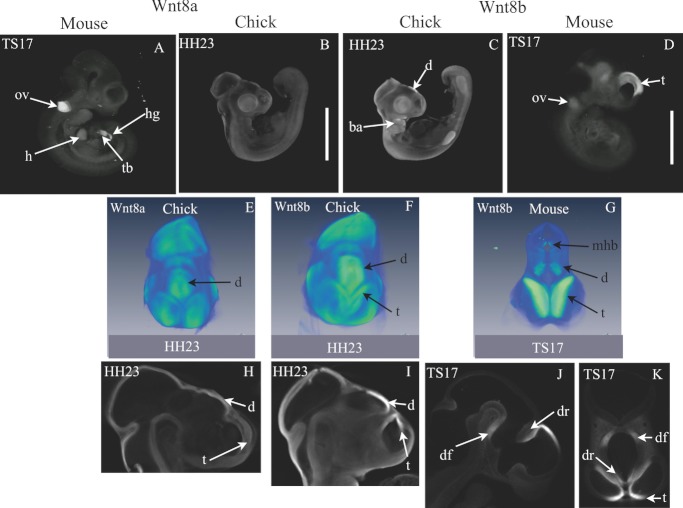
Wnt8 subfamily gene expression in mouse and chick embryos at selected stages TS17 and HH23, as indicated. Mouse Wnt8a is shown in (A); chick Wnt8a in (B, E, and F); chick Wnt8b in (C, F, and I); mouse Wnt8b in (D, G, J, and K). (A–D) show lateral views of the whole embryo and (E–G) frontal views of the head, all external views (volume rendered) following three-dimensional imaging (OPT). (H–K) show sagittal virtual sections through the brain region. Abbreviations: ba, branchial arches; d, diencephalon; df, diencephalon floor; dr, diencephalon roof; h, heart; hg, hindgut; mhb, midbrain-hindbrain boundary; ov, otic vesicle; t, tail; tb, tail bud. Scale bars indicated in (B) and (D) represent 2 mm.

There is very limited expression data on chick Wnt8a in the literature (Hollyday et al. 1995). The data reported here extend previous descriptions in the brain (dorsal telencephalon, diencephalons, and dorsal and ventral midbrain) and report novel sites in the branchial arches and tail bud (supporting information Table S9 and Fig. 8). Chick Wnt8b is also most prominently expressed in the brain but is additionally expressed at low levels in the facial region. Mouse Wnt8a is additionally expressed in limb (in the ventral proximal mesenchyme [TS17] and later in the dorsal ectoderm [TS19]), throughout the otic vesicle, and from TS19 in the hindgut, eye, heart, and tail bud. Mouse Wnt8b shows the well-described (Richardson et al. 1999) and very striking expression in the telencephalon, as well as the anterior diencephalon and the midbrain–hindbrain boundary from TS17, but also additional sites in the eye, otic vesicle, gut, and body wall not seen for the chick orthologue. Although overall the restricted territories in which the paralogues and orthologues are expressed are similar (main sites in CNS, facial region), mouse paralogues show a number of additional territories, often only at later stages of development (TS17 and TS19).

The most notable general feature of all Wnt8 subfamily expression patterns in both mouse and chick is how restricted the patterns are, with chick expression even more restricted than mouse (supporting information Tables S8 and S9 and Figs. 3 and 8). In the chick, outside the facial region and CNS, expression of Wnt8 genes is restricted to Wnt8a in the tail. Limb expression is not a prominent feature of Wnt8 paralogues, unlike the other subfamilies. Comparing Wnt8a orthologues, there are a large number of differences noted due to the number of extra sites expressing Wnt8a in the mouse (Fig. 4 and supporting information Table S10), with brain and tail bud being the only shared sites. It is interesting to note that Wnt8a is also the most coding sequence divergent gene in this study (Fig. 2). Again for the Wnt8b orthologues, there is a large amount of difference in their deployment due to extra sites in the mouse. The only highly similar expression pattern is in the rhombocephalon.

Figure 4 indicates that mouse Wnt8a is expressed in a similar domain to that of Wnt8b in the telencephalon (Fig. 8H), but is only detectable from TS19 and the expression level is very low. Mouse Wnt8b is expressed in two domains of the diencephalon in addition to the very strong expression in the dorsomesial telencephalon; in the roof at the diencephalon–telencephalon boundary and in the floor extending more posteriorly (Fig. 8J and K), (the presumptive hippocampus and hypothalamus, respectively [Fotaki et al. 2010]). Chick Wnt8b is similarly but less-strongly expressed in the dorsomesial telencephalon but is much more prominently expressed in a more extensive domain in the dorsal diencephalon fading toward the midbrain boundary (Fig. 8F and I) and it is not expressed in the floor of the diencephalon; the presumptive hypothalamus (Fig. I). Interestingly, chick Wnt8a is very similarly expressed to chick Wnt8b in the cortical hem, and either side of the dorsal midline in the diencephalon, and is also not expressed in the floor of the diencephalon (Fig. 8E and H). Unlike the mouse paralogues, the chick genes coexpress in these territories from HH20, whereas the mouse genes only coexpress from TS19 and at very different expression levels. So comparing across species, both sets of paralogues have similarities in forebrain expression, however the chick genes are more prominently expressed in the dorsal diencephalon with no expression in the ventral diencephalon whereas mouse genes are more prominently expressed in the telencephalic vesicles.

## DISCUSSION

DNA and protein sequence comparisons show that the developmentally essential Wnt genes are highly conserved, particularly the more recently duplicated paralogous pairs of genes specific to vertebrate lineages. But the coding sequence reflects only one aspect of functional divergence. By carrying out a systematic comparison between the expression domains of paralogous genes within a species (mouse or chick) and the equivalent orthologous genes across species, we have examined another aspect of functional divergence or conservation; the utilization of the gene within the developing embryo, reflecting gene regulation. Comparing the expression patterns of Wnt paralogues in mammalian and avian lineages demonstrates the extent to which embryonic gene deployment has evolved among duplicated genes in each species and between species. In the examples studied here (Wnt2, Wnt5, Wnt7, and Wnt8), the data show a variety of outcomes from paralogues sharing territories of expression, to highly divergent sites of expression. Also across these long-separated species, the domains of some orthologues are very similar and others have entirely diverged. There are some cases of different paralogues (e.g., Wnt2b in the chick and Wnt2 in the mouse) sharing domains that are not shared by the otherwise apparent orthologues (based on coding sequence comparison) indicating that subfunctionalization/neofunctionalization may have occurred differently in the lineages. Within the same pair of paralogues or pair of orthologues, subsets of domains were found to have diverged, consistent with the idea of modular regulation and independent evolution of different regulatory modules. Independent modular regulation was also indicated by an analysis of expression sites that are shared by genes, where groups of shared sites were not found to be reciprocal (Fig. 3B), indicating that either each territory is regulated individually or, even if shared territories are driven in part by shared regulatory modules, the relationship is not simple and the regulation likely to be multifaceted.

**Fig 9:**
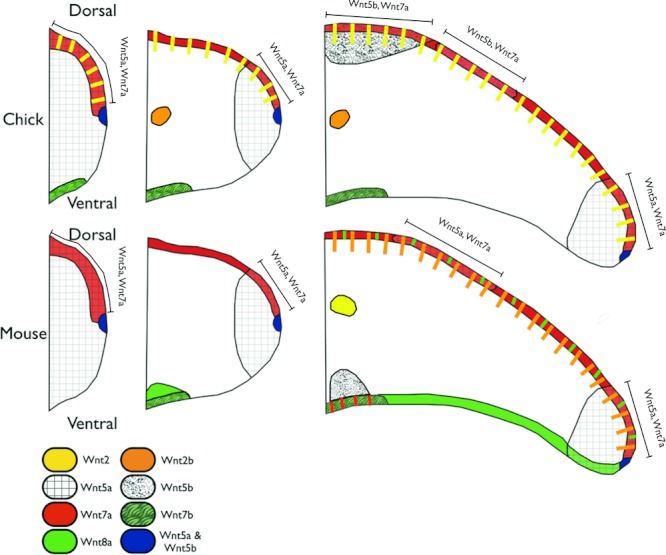
A schematic representation of the expression domains of Wnt2, Wnt5, Wnt7, and Wnt8 paralogous genes during development of the chick (above) and mouse (below) forelimbs. The serial images from left to right approximately represent stages HH20, HH23, and HH26 in the chick; TS15, TS17, and TS19 in the mouse–-shape differences are not faithfully represented. Individual expression domains are represented by color or shading as indicated in the key. Overlapping expression of several genes in the dorsal and proximoventral ectoderm are indicated by superimposed stripes and clarified with labels—for example, in the mouse dorsal ectoderm, Wnt2b, Wnt7a, and Wnt8a are coexpressed; Wnt5a is partially coexpressed; in the chick, Wnt2 and Wnt7a are expressed throughout and Wnt5b is partially coexpressed.

Duplicated genes are considered to diverge through subfunctionalization and/or neofunctionalization, but both processes can occur through evolution of the coding sequences or the regulatory sequences giving distinct and/or novel sites of expression. An interesting question is, to what extent are different Wnt gene products intrinsically functionally distinct and to what extent are they functionally distinct because they are differentially expressed temporally and spatially in the embryo? To examine this, we focused on the developing limb to compare mouse and chick expression domains occupied by each of the eight genes studied here (Fig. 9). It is clear that although the domains of individual genes are not conserved across species, the total sum of expression domains is similar. There are following two exceptions to this: (1) the expression of Wnt5b in a dorsal domain in the proximal chick limb and a ventral domain in the proximal mouse limb, for which there is no corresponding expression of any of this set of genes in the other species. It is possible that a Wnt gene not included in this study is expressed in these territories but for the mouse, where we previously carried out a comprehensive study of all 19 Wnt genes, there is no detectable expression that corresponds to the chick domain of Wnt5b. It is therefore possible that these species-specific domains are related to morphological differences between the species, perhaps related to differential cell proliferation, apoptosis, shape, or movement in these regions. Differences in Wnt1 patterning of the forebrain has been linked to diversity in brain morphology in cichlid fishes (Sylvester et al. 2010). (2) The expression of Wnt8a throughout the surface ectoderm is unique to the mouse; particularly in the ventral ectoderm, this may represent a domain for which there is no equivalent in the chick. All other domains are similar between the species but not the precise family member expressed there. Wnt2 and Wnt2b show reciprocal domains in the two species with Wnt2 expressed in the chick dorsal ectoderm and 2b in the mouse, likewise 2b is expressed in a localized domain of the proximal core mesenchyme in the chick and 2 in a similar domain in the mouse. Wnt5a expression is one of the best characterized in both mouse and chick and shows very similar domains but this is not typical of other genes. Wnt7a expression in the dorsal ectoderm is also well characterized and very similar in both species but the additional, later domain of mouse Wnt7a expression in the ventral proximal ectoderm (Summerhurst et al. 2008) is not seen during the stages covered here in the chick.

Because the gene expression patterns were captured in three-dimensional using similar methodology between the species, the data can be compared and integrated. The patterns were scored for similarity in expression domains, and the integrated data presented in Fig. 4 give an overview of the comparisons between paralogues in each species and orthologues across species for each of the subfamilies. It is clear that the subfamilies that are most highly conserved at the coding sequence level (Fig. 2; Wnt5 and Wnt7) also show greatest similarity in expression domains (although not in all domains). This suggests that the distinct or complementary functional roles of these subfamily paralogues (e.g., Wnt5a vs. Wnt5b) were established early in the common vertebrate ancestor following duplication and have been highly conserved in both lineages, reflected in both the protein products and the domains of expression. This in turn indicates that the functions are essential and fundamental to the general vertebrate body plan. In contrast, Wnt2 and Wnt8 genes have diverged more, indicating they were less constrained to evolve in the different lineages. Mouse Wnt8a in particular shows the lowest level of protein sequence similarity and shows a large number of novel sites of expression.

Presumably differences in regulation between duplicated genes result largely from changes in *cis*-regulatory elements. Although this concept has been supported through many examples, including the recent demonstration of changes in regulatory regions implicated in the evolution of butterfly mimicry (Reed et al. 2011), mapping and comparing regulatory elements between genes is a largely unmet challenge to date. The mouse and chick genomes offer two relatively distant species from parallel lineages for such comparisons and work in this area is now needed to demonstrate the molecular basis of this aspect of functional divergence. Comparative expression analysis, such as that presented here, will be an important prerequisite for such work. The Wnt paralogous genes are very interesting in this respect given their fundamental importance in organizing the animal body plan. To date, there are very few studies that approach the question of expression divergence among closely related Wnt genes (Cox et al. 2010; Beretta et al. 2011). Zebrafish show extra lineage-specific duplications of Wnt genes including four Wnt7 paralogues; the expression patterns were compared by Beretta et al. (2011) who found predominant expression in the CNS across the group, as shown here for the chick and mouse, including distinctive overlapping and complementary domains. They did not report expression in territories homologous to the extra sites we noted for Wnt7 genes in the mouse-–the heart, hindgut, and foregut (Fig. 3 and supporting information Tables S7 and S8). Cho et al. (2010) have compared the expression of four paralogue pairs of duplicated genes in the leech *Helobdella* and with their orthologues in *Capitella* (Wnt5, Wnt11, and Wnt16). They showed clear divergence of paralogues and also found greater similarity in the expression domains of Wnt5 paralogues, with greatest divergence in Wnt11.

The territories in which Wnt7 and Wnt5 subfamilies are expressed show greatest similarity, particularly in the CNS, facial region, and limbs, but the domains within each territory are not identical. In these territories, paralogues often show overlapping or complementary expression and this is conserved, at least in part in the other species. For example, mouse Wnt7a and Wnt7b are widely expressed in the brain but the complementary territories are evident with 7b showing more restricted subdomains (Fig. 7 and supporting information Table S20). The chick genes are expressed in similar domains but the patterns are less complex. All Wnt7 genes are expressed in the neural tube but the paralogues in both species show the same dorso-ventral distinct domains. Similarly, Wnt5 gene expression in the mouse limb ectoderm is complementary with Wnt5a in the dorsal ectoderm and Wnt5b in the proximoventral ectoderm. This distinction is also shared by chick Wnt5 paralogues. It can be hypothesized from this that some key regulatory elements are shared among paralogues and orthologues, directing expression to the CNS territories and limb ectoderm, but that additional sites have led to modification of the precise domains. It is also interesting that expression by any of the genes in any part of the CNS is shared with expression in the mesencephalon indicating a common and perhaps necessary regulatory module. It will be interesting to fully analyze the regulatory elements of these genes to understand the molecular basis of the differential expression and of divergent evolution.

From the protein sequence comparisons (Fig. 2), it was apparent that the “b” paralogues in each case (Wnt2b, Wnt5b, Wnt7b, and Wnt8b) are more similar to the single amphioxus subfamily protein (presumably coincidental that it is consistently the “b” paralogue). However, in the absence of analysis of the ancient common ancestor, it would be impossible to say if the “b” genes are conserved more closely to the ancestral function. One general observation is that the “b” genes tend to show more restricted expression domains than their “a” paralogues in both species. The expression patterns of amphioxus Wnt7, Wnt8, and Wnt5 have been reported. As in the zebrafish, amphioxus Wnt7 was detected predominantly in the CNS (Schubert et al. 2000a) indicating that the expression we see in other sites such as the gut may represent novel sites of expression. Because both Wnt7 paralogues are widely expressed in the CNS of mouse and chick, it is not possible to say if one is more similar to amphioxus domains. Amphioxus Wnt8 is expressed in the forebrain, hindgut, and paraxial mesoderm (Schubert et al. 2000b). It therefore would appear that the ancestral sites are divided between the vertebrate paralogues with Wnt8a and Wnt8b both in the forebrain, Wnt8a reported in early paraxial mesoderm (prior to stages presented here) and Wnt8b in the hindgut (Fig. 3 and supporting information Tables S9 and S10). Additionally, we see both genes expressed in the eye, otic vesicle, heart, and body wall in the mouse, and both Wnt8a orthologues in the tail indicating a substantial number of novel sites compared to amphioxus, particularly in the mouse. Amphioxus Wnt5 expression has previously been compared to vertebrate expression (Schubert et al. 2001). Taking the comparisons together, there is evidence of both subfunctionalization, where territories are divided between the paralogues, and neofunctionalization, where novel sites arise.

This study was limited to relatively late stages of embryonic patterning to allow comparisons in emerging body systems, which may have diverged more, such as the limb. It would be of interest to also examine patterns in early development when any differences might have a profound effect on the overall body plan. Another noteworthy limitation on this work is the difficulty in comparing relative timing of expression. Changes in the relative timing of expression could contribute to heterochrony and influence morphogenesis. Here, we only considered timing in an approximate way by comparing stages at which the overall morphology of the limb is similar (TS15 with HH20, TS17 with HH23, and TS19 with TS26). A systematic comparison of the relative timing of expression could provide valuable information, however this is a big challenge for a number of reasons. Shifts in timing of events in one system or one tissue with respect to another presumably have occurred multiple times in such divergent species. This makes it difficult to establish a framework for a systematic comparison. Some reference points are needed where developmental timing is similar between the species. It would be easier to establish such reference points in more closely related animal groups, and systematic examination of timing variations would be best approached in such cases. Another possibility would be to examine relative timing with respect to the expression of interacting gene products but with such a complex signaling network as Wnt it would be a significant challenge to assemble a critical mass of data on interacting genes. However, this will be facilitated in the future by the growing data sets in the mouse and chick atlases of gene expression, to which this work contributes.

## Supporting information

**Table S1.** Details of gene expression probes. **Table S2.** Wnt sequences retrieved from the Amphioxus and chick genomes together with mouse orthologues (X indicates that this gene is not present in this species). **Table S3.** Wnt2 gene expression in the chick. **Table S4.** Wnt2 gene expression in the mouse. **Table S5.** Wnt5 gene expression in the chick. **Table S6.** Wnt5 gene expression in the mouse. **Table S7.** Wnt7 gene expression in the chick. **Table S8.** Wnt7 gene expression in the mouse. **Table S9.** Wnt8 gene expression in the chick. **Table S10.** Wnt8 gene expression in the mouse. **Table S11.** Comparison of Wnt2 paralogue (Wnt2, Wnt2b) expression in the chick across HH20–HH26 (Similar =, Degree of difference *, **, **, as defined in Methods). **Table S12.** Comparison of Wnt2 paralogue (Wnt2a, Wnt2b) expression in the mouse across TS15–TS19 (Similar =, Degree of difference *, **, ***). **Table S13.** Comparison of Wnt2 orthologue expression in the chick and mouse at stages HH20–HH26 and TS15–TS19, respectively (Similar =, Degree of difference *, **, ***). **Table S14.** Comparison of Wnt2b orthologue expression in the chick and mouse at stages HH20–HH26 and TS15–TS19, respectively (Similar =, degree of difference *, **, ***). **Table S15.** Comparison of Wnt5 paralogue (Wnt5a, Wnt5b) expression in the chick across HH20–HH26 (Similar =, degree of difference *, **, ***). **Table S16.** Comparison of Wnt5 paralogue (Wnt5a, Wnt5b) expression in the mouse across TS15–TS19 (Similar =, degree of difference *, **, ***). **Table S17.** Comparison of Wnt5a orthologue expression in the chick and mouse at stages HH20–HH26 and TS15–TS19, respectively (Similar =, degree of difference *, **, ***). **Table S18.** Comparison of Wnt5b orthologue expression in the chick and mouse at stages HH20–HH26 and TS15–TS19, respectively (Similar =, degree of difference *, **, ***). **Table S19.** Comparison of Wnt7 paralogue (Wnt7a, Wnt7b) expression in the chick across HH20–HH26 (Similar =, degree of difference *, **, ***). **Table S20.** Comparison of Wnt7 paralogue (Wnt7a, Wnt7b) expression in the mouse across TS15–TS19 (Similar =, degree of difference *, **, ***). **Table S21.** Comparison of Wnt7a orthologue expression in the chick and mouse at stages HH20–HH26 and TS15–TS19, respectively (Similar =, degree of difference *, **, ***). **Table S22.** Comparison of Wnt7b orthologue expression in the chick and mouse at stages HH20–HH26 and TS15–TS19, respectively (Similar =, degree of difference *, **, ***). **Table S23.** Comparison of Wnt8 paralogue (Wnt8a, Wnt8b) expression in the chick across HH20–HH26 (Similar =, degree of difference *, **, ***). **Table S24.** Comparison of Wnt8 paralogue (Wnt8a, Wnt8b) expression in the mouse across TS15–TS19 (Similar =, degree of difference *, **, ***). **Table S25.** Comparison of Wnt8a orthologue expression in the chick and mouse at stages TS15–TS19 and HH20–HH26, respectively (Similar =, degree of difference *, **, ***). **Table S26.** Comparison of Wnt8b orthologue expression in the chick and mouse at stages TS15–TS19 and HH20–HH26, respectively (Similar =, degree of difference *, **, ***).
