# Supplementary material for: Differential deployment of paralogous Wnt genes in the mouse and chick embryo during development
Source: Evol Dev. 2012 Mar;14(2):178–95. doi: 10.1111/j.1525-142X.2012.00534.x (PMC3498729; doi:10.1111/j.1525-142X.2012.00534.x)
Supplement: Supplementary file 1 — Table S1. Details of gene expression probes. Table S2. Wnt sequences retrieved from the Amphioxus and chick genomes together with mouse orthologues (X indicates that this gene is not present in this species). Table S3. Wnt2 gene expression in the chick. Table S4. Wnt2 gene expression in the mouse. Table S5. Wnt5 gene expression in the chick. Table S6. Wnt5 gene expression in the mouse. Table S7. Wnt7 gene expression in the chick. Table S8. Wnt7 gene expression in the mouse. Table S9. Wnt8 gene expression in the chick. Table S10. Wnt8 gene expression in the mouse. Table S11. Comparison of Wnt2 paralogue (Wnt2, Wnt2b) expression in the chick across HH20–HH26 (Similar =, Degree of difference *, **, **, as defined in Methods). Table S12. Comparison of Wnt2 paralogue (Wnt2a, Wnt2b) expression in the mouse across TS15–TS19 (Similar =, Degree of difference *, **, ***). Table S13. Comparison of Wnt2 orthologue expression in the chick and mouse at stages HH20–HH26 and TS15–TS19, respectively (Similar =, Degree of difference *, **, ***). Table S14. Comparison of Wnt2b orthologue expression in the chick and mouse at stages HH20–HH26 and TS15–TS19, respectively (Similar =, degree of difference *, **, ***). Table S15. Comparison of Wnt5 paralogue (Wnt5a, Wnt5b) expression in the chick across HH20–HH26 (Similar =, degree of difference *, **, ***). Table S16. Comparison of Wnt5 paralogue (Wnt5a, Wnt5b) expression in the mouse across TS15–TS19 (Similar =, degree of difference *, **, ***). Table S17. Comparison of Wnt5a orthologue expression in the chick and mouse at stages HH20–HH26 and TS15–TS19, respectively (Similar =, degree of difference *, **, ***). Table S18. Comparison of Wnt5b orthologue expression in the chick and mouse at stages HH20–HH26 and TS15–TS19, respectively (Similar =, degree of difference *, **, ***). Table S19. Comparison of Wnt7 paralogue (Wnt7a, Wnt7b) expression in the chick across HH20–HH26 (Similar =, degree of difference *, **, ***). Table S20. Compariso [file ede0014-0178-sd1.pdf]

Supplementary Data Table 1: Details of gene expression probes

Gene	Alignment on cDNA Reference Sequence	Source
Wnt2	549-1009 on XM_416013	RTPCR
Wnt2b	636-1292 on AF182403.1	ChEST598f12
Wnt5a	269-1543 on NM_204887.1	ChEST2k9
Wnt5b	379-1092 on NM_001037269.1	ChEST80a21
Wnt7a	489-1244 on NM_204292.1	ChEST543m22
Wnt7b	933-1334 on NM_001171593	Prof. A McMahon
Wnt8a	1025-1734 on NM_205531	Prof. J.C. Izpisúa Belmonte
Wnt8b	563-954 on XM_426508	Prof. J.C. Izpisúa Belmonte

Supplementary Data Table 2: Wnt sequences retrieved from the Amphioxus and chick genomes together with mouse orthologues (, X indicates that this gene is not present in this species).

Gene	Amphioxus Ref.[1]	Chick Ref.	Ch. location	Mouse Ref.	Ch. location
Wnt1	AF061974	AY655699.1	2:147,605,364-147,640,671	NM_021279.4	15: 98,620,288- 98,624,268
Wnt2	XM_002601713.1	XM_416013.2	1:26,496,353-26,509,804	NM_023653.5	6: 17,938,940- 17,980,585
Wnt2b		NM_204336.1	26: 3,319,520- 3,331,954	NM_009520.3	3: 104,747,723-104,764,627
Wnt3	AF361013	NM_001081696.1	27	NM_009521.2	11: 103,635,464-103,679,271
Wnt3a		NM_001171601.1	2: 2,363,709- 2,451,014	NM_009522.2	11: 59,061,535- 59,104,254
Wnt4	AF061973	NM_204783.1	21: 6,505,909- 6,517,953	NM_009523.1	4: 136,833,404-136,855,641
Wnt5a	AF361014_1	NM_204887.1	12: 8,133,521- 8,145,608	NM_009524.2	14: 29,317,936- 29,340,633
Wnt5b		NM_001037269.1	1: 63,037,409- 63,053,152	NM_009525.3	6: 119,382,549-119,494,336
Wnt6	AF361015	NM_001007594.1	7: 23,961,932- 23,965,054	NM_009526.3	1: 74,818,466- 74,831,893
Wnt7a	XM_002597242.1	NM_204292.1	12: 6,244,431-6, 277,634	NM_009527.3	6: 91,313,975- 91,361,357
Wnt7b		NM_001037274.1	1: 72,939,329- 73,031,977	NM_001163633	15: 85,365,869- 85,412,251
Wnt8a	AF190470	NM_205531.1	13: 14,563,742-14,567,673	NM_009290.2	18: 34,701,967- 34,707,927
Wnt8b		XM_426508.2	6: 18,535,630- 18,541,789	NM_011720.3	19: 44,567,962- 44,588,763
Wnt9a	fgenesh2_pg.scaffold_12000179	NM_204981.1	2: 2,483,585- 2,535,827	NM_139298.2	11: 59,120,430- 59,147,054
Wnt9b		XM_001234393.1	27: 1,071,170- 1,081,932	NM_011719.4	11: 103,588,678-103,611,135
Wnt10a	fgenesh2_pg.scaffold_1068000002	NM_001006590.1	7: 23,942,411- 23,952,897	NM_009518.2	1: 74,838,090- 74,850,753
Wnt10b		X	X	NM_011718.2	15: 98,602,184- 98,608,581
Wnt11	AF187533	NM_204784.1	1: 198,826,726-198,854,182	NM_009519.2	7: 105,983,622-106,003,705
Wnt11b		NM_001130744.1	4:1,181,747-1,183,797	X	X
Wnt16	fgenesh2_pg.scaffold_27000091	XM_001233677.1	1: 25,034,205- 25,044,964	NM_053116.4	6: 22,238,227- 22,250,409

1: Genbank reference where available or Amphioxus genome reference

Supplementary Data Table 3: Wnt2 Gene Expression in the Chick.
Overview of gene expression patterns of Wnt2 and Wnt2b in chick embryos at stages HH20, HH23 and HH26.

Chick	System	Wnt2	Wnt2b
HH20	CNS	telencephalon, dorsolateral patches; diencephalon, surrounding but excluding midline of dorsal; midbrain, throughout dorsal; rhombocephalon, dorsal	diencephalon, restricted both sides of the dorsal midline and extending laterally into prosomere 2, excluding the zona limitales; midbrain, dorsal midline graded anterior to posterior, restricted to ventricular margin posteriorly, midbrain side of midbrain/hindbrain boundary
	Facial	branchial arch 1, diffuse in maxillary component, distal and graded anterior to posterior in mandibular component; branchial arch 2, posterior with highest levels proximally	branchial arches, superficial in all four; branchial arch 1, highest level in anteriorly mesial region of mandibular component
	Limb	dorsal mesenchyme and ectoderm	
	Eye	optic cup	corneal epithelium, lateral tips of optic cup and lateral edges of lens vesicle
	Otic	throughout, highest dorsally	restricted dorsally
	Heart	pericardium	
	Lung		lung buds
	Foregut		extensive between level posterior to branchial arch 4 and forelimb; oesophagus most dorsal and most ventral aspects
	Hindgut	diffuse low levels	hindgut diverticulum, localized at level of hindlimb buds
	Body wall		throughout body wall
HH23	CNS	as HH20, dorsal ectoderm overlying neural tube	As HH20, diencephalon, prosomere 2 domain now oriented mesially forming a ‘sickle’ pattern either side of midline
	Facial	branchial arch 1 and branchial arch 2 low levels diffuse distally	As HH20 except; also frontonasal process and expression extends laterally and deeper in anterior mandibular branchial arch 1
	Limb	as HH20	Very localized mesenchyme at proximal core of all limb buds, dorsal of midline; hindlimb also overlying ectoderm
	Eye	as HH20	As HH20
	Otic		Localized to endolymphatic appendage and coclear duct
	Heart	as HH20	
	Lung		As HH20
	Foregut		As HH20
	Hindgut		As HH20
	Body wall		Down regulated but detected in base of amnion
HH26	CNS	as HH23	telencephalon, low levels; midbrain down regulated; diencephalon, still detectable in prosomere 2 but not as defined
	Facial	Frontonasal processes diffuse, branchial arch 1 and branchial arch 2, low levels diffuse laterally and extending dorsally onto flank	branchial arches; downregulated, still detectable in posterior and distal extremities
	Limb	as HH23	As HH23 with hindlimb surface ectoderm more extensive

Chick	System	Wnt2	Wnt2b
	Eye	as HH23	as HH23
	Otic		endolymphatic appendage
	Heart	as HH23	
	Lung		As HH23
	Foregut		Down regulated, more diffuse
	Hindgut		As HH23
	Body wall		Base of amnion
	Tail	dorsal tail bud	

Supplementary Data Table 4: Wnt2 Gene Expression in the Mouse.
Overview of gene expression patterns of Wnt2 and Wnt2b in mouse embryos at stages TS15, TS17 and TS19.

**Mouse**	**System**	**Wnt2**	**Wnt2b**
TS15	CNS		diencephalon, throughout; midbrain, weak expression in dorsal midline of anterior half
	Heart	atria and associated vasculature	
	Foregut	extensive ventrally	
	Hindgut		hindgut diverticulum
	Body wall	throughout body wall, umbilicus and future placenta	
TS17	CNS		midbrain, weak in dorsal midline; diencephalon, weak in dorsal midline
	Eye	lateral tips of optic cup, especially posteriorly	throughout optic cup
	Otic	throughout and endolymphatic appendage	throughout and endolymphatic appendage
	Heart	low level	
	Lung	lung bud mesenchyme	
	Foregut	dorsal and ventral foregut from posterior to branchial arch 4 to level of forelimb	
	Hindgut		highly downregulated and restricted ventrally
	Body wall	as TS15	
TS19	CNS		midbrain, throughout dorsal midline; diencephalon, throughout dorsal midline
	Facial	superficial distal patches on branchial arch 1 and 2	superficial distal patches on frontonasal process, branchial arch 1 and 2 (more extensive than Wnt2)
	Limb	mesenchymal patch, proximal core	dorsal ectoderm and adjacent mesenchyme
	Eye	corneal epithelium	as TS17
	Otic	as TS17	as TS17
	Lung	as TS17	
	Foregut	restricted ventrally	
	Body wall	as TS17	

Supplementary Data Table 5: Wnt5 Expression in the Chick.
Overview of gene expression patterns of Wnt5a and Wnt5b in mouse embryos at TS15, TS17 and TS19.

Chick	System	Wnt5a	Wnt5b
HH20	CNS	diencephalon, dorsal midline in sickle shape excluded from midline, ventral regions on posterior domain; midbrain, restricted ventral pattern, extensive anteriorly, posteriorly excluded from midline; localised regions lateral to midline extending into diencephalon; rhombencephalon, low levels along ventral midline	telencephalon, between vesicles and in a broad pattern anteriorly mesially; diencephalon, dorsally; midbrain, ventricular aspect localised excluded from midline and adjacent region, midbrain-hindbrain boundary diffuse
	Facial	branchial arch 1, maxillary component low levels diffuse, mandibular component lateral mesenchyme distally; branchial arch 2, 3 and 4 mid-distal lateral mesenchyme	branchial arches in core mesenchyme only; branchial arch 1, maxillary component of diffuse in proximal region
	Limb	graded in mesenchyme with higher levels toward the posterior with even higher levels in the AER	AER; restricted in dorsal proximal limb running in to body wall
	Eye	optic cup; cornea	lens, cornea
	Otic Vesicle		mesenchyme posterior to
	Heart	throughout ventricles	
	Lung	bud mesenchyme	
	Foregut	at diverticulum and adjacent mesenchyme	at level of branchial arch 4
	Midgut	restricted	
	Hindgut		hindgut diverticulum
	Somites		restricted to the dorsal most and the ventral most aspect
	Tail	tail bud; mesoderm of tail low level extensive	tail bud; ectoderm, mesoderm of tail, more extensive
HH23	CNS	as for HH20	as for HH20; midbrain, localised in posterior ventricular region; rhombocephalon, ventral
	Facial	as for HH20; Frontonasal process diffuse; endoderm of third cleft	branchial arch 1, maxillary component highest levels anteriorly, mandibular component diffuse but highest distally; branchial arch 2, highest levels posteriorly distally and laterally, branchial arch 3, diffuse; branchial arch 4 diffuse
	Limb	expression is restricted to the more distal part of the limb but retains a similar pattern in the AER and the posterior part of the distal limb	as HH20
	Eye	lateral edges of the eye; cornea	as HH20
	Otic Vesicle		as HH20
	Heart	pericardium	
	Lung	mesenchyme of buds and of trachea	
	Foregut	mesenchyme around dorsal anterior foregut at level of oesophagus	
	Somites	restricted to the dorsal most and the ventral most aspect	
	Tail	as HH20	as HH20
HH26	CNS	diencephalon, midline; midbrain, broad ventrally, midbrain-hindbrain boundary; rhombencephalon ventral	as for HH23 but extended and higher levels in telencephalon

Chick	System	Wnt5a	Wnt5b
	Facial	frontonasal process, highest levels distally; branchial arch 1 maxillary and mandibular component restricted	branchial arch 2 posterior localised; branchial arch 3 and 4 at cleft pouches
	Limb	as HH23; additionally in posterior and anterior proximal hindlimb	as HH23 but expression in proximal mesenchyme extends distally in a skeletal pattern
	Eye	optic cup; corneal	As HH23; optic cup
	Otic vesicle		throughout posterior mesenchyme and flank below otic vesicle at level of branchial arches
	Somites	dermamyotome	
	Body wall	lateral line	lateral line
	Tail	as HH23	highest levels in ectoderm and diffuse in mesenchyme

Supplementary Data Table 6: Wnt5 Expression in the Mouse.
Overview of gene expression patterns of Wnt5a and Wnt5b in mouse embryos at TS15, TS17 and TS19.

Mouse	System	Wnt5a	Wnt5b
TS15	CNS	telencephalon, ventrally; diencephalon, posterior ventrally; midbrain, anterior ventrally and posterior dorsally; rhombencephalon, rhombomere 1; neural tube, ventral aspect extending posteriorly to level of forelimb	midbrain, ventrally
	Facial	frontonasal process, throughout, branchial arch 1 mandibular component gradient from anterior to posterior and lateral to medial; branchial arch 2 in a gradient from anterior to posterior and lateral to medial; branchial arch 3, diffuse	
	Limb	distal mesenchyme and AER	
	Otic Vesicle	low levels in dorsal posterior aspect	
	Lung	throughout lung bud mesenchyme	
	Somites	dermamyotome, graded anterior to posterior	
	Body Wall	lateral body wall, restricted at level of lung buds	
	Tail	tail bud; mesoderm, extensive; hindgut; neural plate	
TS17	CNS	midbrain, ventrally; rhombocephalon, anterior ventrally, ventricular zone from rhombomere 5 into anterior neural tube	as TS15 but more restricted
	Facial	frontonasal process, ventral; branchial arch 1, distal mesenchyme of maxillary component, anterior mesially in mandibular component; branchial arch 2, posteriorly, branchial arch 3, diffuse; branchial arch 4, diffuse	frontonasal process, throughout; branchial arch 1, distal mesenchyme of maxillary component, anterior mesially of mandibular component; branchial arch 2, posterior laterally; branchial arch 3, diffuse; branchial arch 4, strong in endoderm of 4th cleft
	Limb	as TS15	
	Otic Vesicle	as TS15	otic vesicle and endolymphatic appendage
	Lung	as TS15	
	Foregut	anterior foregut	
	Hindgut	diverticulum, ventrally low levels	diverticulum, ventrally
	Somites	sclerotome	
	Body Wall	as TS15	
	Tail	as TS15	tail bud; ectoderm; mesoderm, restricted; neural ectoderm
TS19	CNS	telencephalon, ventrally and cortical hem; midbrain, broad ventrally, midbrain-hindbrain boundary; rhombencephalon, ventrally; (mainly floor apart from boundaries)	as TS17; rhombencephalon, ventral
	Facial	frontonasal process, distal aspect of olfactory pit; branchial arch 1, maxillary component diffuse but excluded from core, mandibular component distally, anterior mesially; branchial arch 2, diffuse; branchial arch 3, diffuse; branchial arch 4, diffuse	frontonasal process, diffuse; branchial arches, diffuse

Mouse	System	Wnt5a	Wnt5b
	Limb	as TS17; mid-limb dorsal mesenchyme	ventral proximal mesenchyme
	Eye	optic cup	
	Otic Vesicle	throughout	as TS17
	Lung	as TS17	
	Foregut	stomach wall, anteriorly restricted	foregut mesenchyme in pharyngeal region; mesenchyme of stomach and duodenum
	Somites	dermamyotome of top two somites	sclerotome of posterior somites highest levels dorsally
	Body Wall	lateral line; lateral body wall	
	Tail	as TS17	
	Genitourinary	throughout	throughout

Supplementary Data Table 7: Wnt7 Expression in the Chick.
An overview of the gene expression patterns of Wnt7a and Wnt7b in the chick across HH20, HH23 and HH26.

Chick	System	Wnt7a	Wnt7b
HH20	CNS	telencephalon, laterally; diencephalon, dorsal middle anteriorly; midbrain, lateral and ventral; rhombocephalon, ventral; neural tube, ventral midline (and notochord)	telencephalon, throughout dorsal posterior region; diencephalon, midline and lateral ventrally; midbrain dorso-lateral and ventral, midbrain-hindbrain boundary; rhombencephalon, ventral; neural tube, ventrally with low levels posteriorly
	Facial	branchial arch 1,3 and 4, posterior dorsal domain, very restricted proximally; branchial arch 2, extended full length of branchial arch posteriorly	
	Limb	dorsal ectoderm and surrounding mesenchyme	proximal ventral ectoderm
	Eye	optic cup	optic cup, cornea, lens
	Otic vesicles	throughout	throughout
	Lung		mesenchyme
	Somites	at level between limbs	
HH23	CNS	midbrain, lateral; rhombencephalon, as HH20; neural tube, as HH20	telencephalon, diffuse; diencephalon, ventral broad; midbrain, ventral broad; neural tube, ventral
	Facial	as HH20	
	Limb	as HH20, restricted to ectoderm only	as HH20
	Eye	as HH20; cornea	as HH20; not in lens
	Otic vesicles		as HH20
HH26	CNS	as HH23 extended more dorsally in midbrain, no longer detected in notochord	telencephalon, as HH20; diencephalon, dorsal; midbrain dorsal and ventral broad, midbrain-hindbrain boundary
	Facial		frontonasal process, diffuse; branchial arches, diffuse, highest distally
	Limb	as HH23	as HH23
	Eye	cornea	as HH23
	Otic vesicles	as HH20	as HH23
	Lung		as HH20
	Midgut		restricted

Supplementary Data Table 8: Wnt7 Gene Expression in the Mouse.
Overview of gene expression patterns of Wnt7a and Wnt7b in chick embryos at stages TS15, TS17 and TS19.

Mouse	System	Wnt7a	Wnt7b
TS15	CNS	telencephalon, lateral vesicles; diencephalon, ventral domain restricted laterally; midbrain, broad dorso-lateral; rhombocephalon, ventral; neural tube, ventral domain to level of forelimb	telencephalon, surrounding but excluded from the midline, dorsal domain broad posteriorly then tapering mesially anteriorly; diencephalon, either side of midline in prosmere 1, midbrain, posterior ventral aspect, extending slightly across the ventral aspect in a semi-circle and about one third of the way up the midline dorsally; neural tube, ventral very localised region just posterior to otic vesicles
	Facial	branchial arches, diffuse, highest levels laterally	ectoderm of all branchial arches except maxillary component
	Limb	dorsal ectoderm and surrounding mesenchyme	throughout ectoderm
	Eye		ventral edge, optic stalk
	Otic vesicle	throughout	throughout, highest levels dorsally
	Lung		mesenchyme
	Foregut	low levels	
	Hindgut	diverticulum	throughout
TS17	CNS	as TS15 but midbrain, lateral mostly ventral lateral stripes more dorsal in the posterior region; neural tube, dorsal part of ventral domain	telencephalon, as TS15 in dorsal domain; diencephalon, lateral, ventral; rhomocephalon, ventral, diffuse anteriorly then becomes very defined; neural tube, ventral
	Facial		branchial arch 1, ectoderm of mandibular component; branchial arch 2, ectoderm of mandibular component;
	Limb	as TS15 but restricted to ectoderm only	as TS15 but restricted to proximal ventral ectoderm
	Eye		two patches of expression laterally on posterior edge
	Otic vesicle		as TS15
	Lung		as TS15
TS19	CNS	as TS17 but diencephalon, posterior lateral mostly ventral, midbrain, lateral extending dorsally as move posterior; neural tube, ventrally throughout	as TS17 but diencephalon, ventral, anterior dorsal patches excluding midline in prosmere 1, lateral ventral patches in prosmere 2; midbrain, ventral; neural tube, ventrally throughout
	Facial	branchial arches, ectoderm; frontonasal process, ectoderm	branchial arches, ectoderm; frontonasal process, ectoderm
	Limb	ventral proximal ectoderm, dorsal handplate ectoderm excluding distal edges	as TS17
	Eye		as TS17
	Otic vesicle	as TS15 with highest levels dorsally	as TS17
	Heart	pericardium	
	Body wall		ectoderm of body wall
	Lung		as TS17

Supplementary Data Table 9: Wnt8 Expression in the Chick.
Overview of gene expression patterns of Wnt8a and Wnt8b in mouse embryos at TS15, TS17 and TS19.

Chick	System	Wnt8a	Wnt8b
HH20	CNS	telencephalon, dorsal anteriorly; diencephalon, dorsal; midbrain, dorsal and ventral	telencephalon, vesicles dorsally; diencephalon either side of dorsal midline (stripes); midbrain, dorsal; rhombocephalon, ventral
	Facial	branchial arches, diffuse throughout all, highest in branchial arch 1	frontonasal process, diffuse; branchial arches, diffuse
	Tail	tip of tail bud	
HH23	CNS	as HH20 but not in ventral midbrain	as HH20
	Facial	as HH20 but lower levels	as HH20, branchial arches, highest levels distally
	Tail	as HH20	
HH26	CNS	as HH23	as HH23
	Facial	as for HH20, frontonasal process diffuse	as HH23

Supplementary Data Table 10: Wnt8 Expression in the Mouse.
An overview of the gene expression pattern of Wnt8a and Wnt8b in the mouse at TS15, TS17 and TS19.

Mouse	System	Wnt8a	Wnt8b
TS15	CNS		telencephalon, dorsal posterior and mesial; diencephalon, anteriorly either side of midline dorsally
TS17	CNS	diencephalon, ventral	telencephalon, dorsal posterior and mesial but excluded from midline; diencephalon, ventral; midbrain-hindbrain boundary
	Facial	branchial arches, diffuse throughout all, highest levels anterior and mesially on mandibular component of branchial arch 1	
	Limb	ventral proximal mesenchyme	
	Eye		optic cup
	Otic vesicle	throughout	throughout and mesenchyme dorsally
	Heart		diffuse
	Midgut		diffuse
	Hindgut	high levels throughout	
	Body wall		anterior lateral line (from otic vesicle to forelimb) and lateral body wall
TS19	CNS	telencephalon, posterior vesicles tapering mesially; diencephalon, localised patch dorsal anterior; midbrain, ventral midbrain hindbrain boundary	as TS17, extended to ventral rhombocephalon
	Facial	branchial arches, diffuse; frontonasal processes, diffuse	
	Limb	throughout surface ectoderm	
	Eye	lens vesicle, optic cup	
	Otic vesicle	as TS17	
	Heart	atria	
	Body wall	ectoderm	
	Tail	throughout tip	

Supplementary Data Table 11: Comparison of Wnt2 Paralogue (Wnt2,Wnt2b) Expression in the Chick across HH20-HH26 (Similar =, Degree of difference ✶, ✶✶, ✶✶✶, as defined in Methods).

**Chick**	**System**	**Wnt2**	**Wnt2b**	**Similarity/ Difference**
**CNS**	Telencephalon	✓ dorsolateral patches	✓ low levels from HH26	✶✶
	Diencephalon	✓ surrounding but excluding midline	✓ surrounding but excluding midline, more restricted	✶
	Midbrain	✓ dorsal	✓ dorsal midline	✶
	MHB	X	✓	✶✶✶
	Rhombocephalon	✓	X	✶✶✶
**Facial**	Branchial arches	✓ branchial arch 1 and branchial arch 2, complex	✓ superficial in all	✶✶
	Frontonasal Process	✓	✓	=
**Limb**	Mesenchyme	✓ dorsal	✓ localized mesenchyme at proximal core of all limb buds	✶✶
	Ectoderm	✓	X	✶✶✶
**Other**	Eye	✓ optic cup	✓ optic cup, lateral tips; corneal epithelium, lateral edges of lens vesicle	✶✶
	Otic	✓ throughout, highest dorsally	✓ dorsal; endolymphatic appendage; coclear duct	✶
	Heart	✓	X	✶✶✶
	Lung	X	✓	✶✶✶
	Foregut	X	✓	✶✶✶
	Hindgut	✓ diffuse low levels	✓ hindgut diverticulum	✶
	Body wall	X	✓	✶✶✶
	Tail	✓	X	✶✶✶

Supplementary Data Table 12: Comparison of Wnt2 Paralogue (Wnt2a,Wnt2b) Expression in the Mouse across TS15-TS19 (Similar =, Degree of difference ∗, ∗∗, ∗∗∗).

Mouse	System	Wnt2	Wnt2b	Similar/ Different
**CNS**	Diencephalon	X	✓	∗∗∗
	Midbrain	X	✓	∗∗∗
**Facial**	Branchial Arches	✓	✓	=
	Frontonasal Processes	X	✓	∗∗∗
**Limb**	Mesenchyme	✓ proximal core	✓ dorsal	∗∗
	Ectoderm	X	✓ dorsal	∗∗∗
**Other**	Eye	✓ optic cup, lateral tips; corneal epithelium	✓ optic cup, throughout	∗∗
	Otic Vesicle	✓	✓	=
	Heart	✓	X	∗∗∗
	Lung	✓	X	∗∗∗
	Foregut	✓	X	∗∗∗
	Hindgut	X	✓	∗∗∗
	Body Wall	✓	X	∗∗∗

Supplementary Data Table 13: Comparison of Wnt2 Orthologue Expression in the Chick and Mouse at stages HH20-HH26 and TS15-TS19 respectively (Similar =, Degree of difference ✶, ✶✶, ✶✶✶).

	System	Chick Wnt2	Mouse Wnt2	Similar/ Different
**CNS**	Telencephalon	✓	X	✶✶✶
	Diencephalon	✓ surrounding but excluding midline	X	✶✶✶
	Midbrain	✓ dorsal	X	✶✶✶
	Rhombocephalon	✓	X	✶✶✶
**Facial**	Branchial Arches	✓ branchial arch 1 and branchial arch 2	✓ superficial distal patches on branchial arch 1 and 2 from TS19	✶
	Frontonasal Processes	✓	X	✶✶✶
**Limb**	Mesenchyme	✓ dorsal	✓ proximal core, from TS19	✶✶
	Ectoderm	✓	X	✶✶✶
**Other**	Eye	✓ optic cup	✓ corneal epithelium	✶✶
	Otic Vesicle	✓ throughout, highest dorsally	✓ throughout; endolymphaic appendage	✶✶
	Heart	✓ pericardium	✓ atria and associated vasculature	✶✶
	Lung	X	✓	✶✶✶
	Foregut	X	✓	✶✶✶
	Hindgut	✓	X	✶✶✶
	Body Wall	X	✓	✶✶✶
	Tail	✓	X	✶✶✶

Supplementary Data Table 14: Comparison of Wnt2b Orthologue Expression in the Chick and Mouse at stages HH20-HH26 and TS15-TS19 respectively (Similar =, Degree of difference ✶, ✶✶, ✶✶✶).

Stage	System	Chick Wnt2b	Mouse Wnt2b	Similarity/ Difference
**CNS**	Telencephalon	✓	X	✶✶✶
	Diencephalon	✓ 'sickle' pattern either side of midline	✓throughout then dorsal midline	✶✶
	Midbrain	✓ dorsal midline, complex	✓ dorsal midline	✶
	MHB	✓	X	✶✶✶
**Facial**	Branchial Arches	✓ superficial in all	✓ superficial distal patches on branchial arch 1 and 2 from TS19	✶✶
	Frontonasal processes	✓ throughout from HH23	✓ superficial distal patches from TS19	✶✶
**Limb**	Mesenchyme	✓ proximal core	✓ dorsal from TS19	✶✶
	Ectoderm	X	✓ dorsal	✶✶✶
**Other**	Eye	✓ optic cup, lateral tips; lens vesicle, lateral edges, corneal epithelium,	✓ optic cup, throughout	✶✶
	Otic Vesicle	✓ throughout; endolymphatic appendage	✓ throughout; endolymphatic appendage	=
	Lung	✓	X	✶✶✶
	Foregut	✓	X	✶✶✶
	Hindgut	✓	✓	=
	Body wall	✓	X	✶✶✶

Supplementary Data Table 15: Comparison of Wnt5 Paralogue (Wnt5a,Wnt5b) Expression in the Chick across HH20-HH26 (Similar =, Degree of difference ✶, ✶✶, ✶✶✶).

Chick	Region	Wnt5a	Wnt5b	Similarity/ Difference
**CNS**	Telencephalon	X	✓	✶✶✶
	Diencephalon	✓ ventral, dorsal	✓ dorsal	✶✶
	Midbrain	✓ ventral, complex	✓ ventral	✶
	MHB	✓	✓	=
	Rhombocephalon	✓	✓ from HH23	✶
**Facial**	Branchial Arches	✓ distal	✓ core and distal	✶
	Frontonasal Processes	✓	X	✶✶✶
**Limb**	Mesenchyme	✓ distal	✓ dorsal proximal from HH23, dorsal midline from HH25	✶✶
	Ectoderm, AER	✓	✓	=
**Other**	Eye	✓ optic cup, cornea, lateral edges	✓ optic cup, cornea, lens	✶✶
	Otic vesicle	X	✓	✶✶✶
	Heart	✓	X	✶✶✶
	Lung	✓	X	✶✶✶
	Foregut	✓ extensive	✓ level of branchial arch 4	✶
	Midgut	✓	X	✶✶✶
	Hindgut	X	✓	✶✶✶
	Body wall	✓	✓	=
	Somites	✓dorsal and ventral extremities, HH23, dermamyotome HH26	✓ dorsal and ventral extremities, HH20 only	✶✶
	Tail	✓ mesenchyme	✓ mesenchyme and ectoderm	✶

Supplementary Data Table 16: Comparison of Wnt5 Paralogue (Wnt5a,Wnt5b) Expression in the Mouse across TS15-TS19 (Similar =, Degree of difference ✶, ✶✶, ✶✶✶).

Mouse	Region	Wnt5a	Wnt5b	Similarity/ Difference
**CNS**	Telencephalon	✓	X	✶✶✶
	Diencephalon	✓	X	✶✶✶
	Midbrain	✓ TS15 anterior ventral, TS17 posterior dorsal, TS19 ventral	✓ ventral	✶✶
	MHB	✓	X	✶✶✶
	Rhombencephalon	✓ ventral from TS15	✓ ventral from TS19	✶
	Neural tube	✓	X	✶✶✶
**Facial**	branchial arch	✓ complex	✓ complex	✶
	frontonasal processes	✓	✓ from TS17	✶
**Limb**	mesenchyme	✓ distally; TS19 mid-limb dorsally	✓ ventral proximal from TS19	✶✶
	ectoderm, AER	✓	X	✶✶✶
**Other**	Eye	✓ optic cup	✓ optic cup; lens	✶✶
	Otic vesicle	✓ dorsal posterior; throughout from TS19	✓ throughout; endolymphatic apppendage	✶✶
	Lung	✓	X	✶✶✶
	Foregut	✓ from TS17	✓ from TS19	✶
	Body wall	✓	X	✶✶✶
	Somites	✓ dermamyotome TS15, 19; sclerotome TS17	✓ sclerotome at TS19	✶✶
	Tail	✓ mesenchyme	✓ mesenchyme and ectoderm	✶
	Genitourinary	✓	✓	=

Supplementary Data Table 17: Comparison of Wnt5a Orthologue Expression in the Chick and Mouse at stages HH20-HH26 and TS15-TS19 respectively (Similar =, Degree of difference ✶, ✶✶, ✶✶✶).

System	Region	Chick Wnt5a	Mouse Wnt5a	Similarity/ Difference
**CNS**	Telencephalon	X	✓	✶✶✶
	Diencephalon	✓ ventral, dorsal	✓ ventral	✶✶
	Midbrain	✓ ventral, complex	✓ TS15 anterior ventral, TS17 posterior dorsal, TS19 ventral,	✶✶
	MHB	✓	✓	=
	Rhombocephalon	✓	✓	=
	Neural tube, ventral	X	✓	✶✶✶
**Facial**	branchial arches	✓ distal	✓ complex	✶
	frontonasal processes	✓	✓	=
**Limb**	mesenchyme	✓ distal	✓ distally, TS19 mid-limb dorsally	✶✶
	ectoderm, AER	✓	✓	=
**Other**	Eye	✓ optic cup; cornea; lateral edges	✓ optic cup	✶✶
	Otic vesicle	X	✓	✶✶✶
	Heart	✓	X	✶✶✶
	Lung	✓	✓	=
	Foregut	✓ throughout, from TS17	✓ anterior, stomach wall from TS17	✶
	Midgut	✓ throughout	X	✶✶✶
	Hindgut	X	✓	✶✶✶
	Body wall	✓ lateral line	✓ lateral line and level of lung buds	✶✶
	Somites	✓dorsal and ventral extremities, HH23, dermamyotome HH26	✓ dermamyotome TS15, 19; sclerotome TS17	✶✶
	Tail	✓	✓	=
	Genitourinary	X	✓	✶✶✶

Supplementary Data Table 18: Comparison of Wnt5b Orthologue Expression in the Chick and Mouse at stages HH20-HH26 and TS15-TS19 respectively (Similar =, Degree of difference ✶, ✶✶, ✶✶✶).

System	Region	Chick Wnt5b	Mouse Wnt5b	Similarity/ Difference
**CNS**	Telencephalon	✓	X	✶✶✶
	Diencephalon	✓	X	✶✶✶
	Midbrain	✓	✓	=
	MHB	✓	X	✶✶✶
	Rhombocephalon	✓	✓	=
**Facial**	branchial arches	✓ core and distal	✓ complex	✶
	frontonasal processes	X	✓	✶✶✶
**Limb**	mesenchyme	✓ dorsal proximal from HH23, dorsal midline from HH25	✓ ventral proximal from TS19	✶✶
	ectoderm, AER	✓	X	✶✶✶
**Other**	Eye	✓ optic cup; cornea; lens vesicle	✓ optic cup; lens	✶✶
	Otic vesicle	✓ throughout	✓ throughout; endolymphatic apppendage	✶✶
	Foregut	✓ at branchial arch 4	✓ anterior, from TS19	✶✶
	Hindgut	✓ throughout	✓ diverticulum	✶
	Body wall	✓	X	✶✶✶
	Somites	✓ dorsal and ventral extremities, HH20 only	✓ sclerotome at TS19	✶✶
	Tail	✓	✓	=
	Genitourinary	X	✓	✶✶✶

Supplementary Data Table 19: Comparison of Wnt7 Paralogue (Wnt7a,Wnt7b) Expression in the Chick across HH20-HH26 (Similar =, Degree of difference ✶, ✶✶, ✶✶✶).

**Chick**	**Region**	**Wnt7a**	**Wnt7b**	**Similarity/ Difference**
**CNS**	Telencephalon	✓ lateral	✓ dorsal posterior	✶✶
	Diencephalon	✓ dorsal	✓ ventral at HH20,23; dorsal at HH26	✶✶
	Midbrain	✓ lateral and ventral	✓ dorsolateral and ventral	✶✶
	MHB	X	✓	✶✶✶
	Rhombencephalon	✓	✓	=
	Neural tube	✓ ventral midline	✓ ventral	✶
**Facial**	Branchial arch	✓ posterior, restricted	✓ distal	✶✶
	Frontonasal processes	X	✓	✶✶✶
**Limb**	Mesenchyme	✓	X	✶✶✶
	Ectoderm	✓ dorsal	✓ throughout, proximal ventral	✶✶
**Other**	Eye	✓ optic cup; cornea	✓ optic cup; cornea; lens	✶✶
	Otic vesicle	✓	✓	=
	Lung	X	✓	✶✶✶
	Midgut	X	✓	✶✶✶
	Somites	✓	X	✶✶✶

Supplementary Data Table 20: Comparison of Wnt7 Paralogue (Wnt7a,Wnt7b) Expression in the Mouse across TS15-TS19 (Similar =, Degree of difference ✶, ✶✶, ✶✶✶).

Mouse	Region	Wnt7a	Wnt7b	Similarity/ Difference
**CNS**	Telencephalon	✓ lateral	✓ dorsal	✶✶
	Diencephalon	✓ ventral and lateral	✓ ventral and lateral, more complex	✶
	Midbrain	✓ dorsolateral	✓ dorsal and ventral, complex	✶✶
	Rhombencephalon	✓ ventral	✓ diffuse anteriorly, defined posteriorly	✶
	Neural tube	✓	✓	=
**Facial**	Branchial arch	✓ throughout	✓ ectoderm	✶
	Frontonasal processes	✓	✓	=
**Limb**	Mesenchyme	✓	X	✶✶✶
	Ectoderm	✓ dorsal, proximal ventral	✓ throughout, proximal ventral	✶
**Other**	Eye	X	✓	✶✶✶
	Otic vesicle	✓	✓	=
	Heart	✓	X	✶✶✶
	Lung	X	✓	✶✶✶
	Foregut	✓	X	✶✶✶
	Hindgut	✓ diverticulum	✓ throughout	✶
	Body wall	X	✓	✶✶✶

Supplementary Data Table 21: Comparison of Wnt7a Orthologue Expression in the Chick and Mouse at stages HH20-HH26 and TS15-TS19 respectively (Similar =, Degree of difference ✶, ✶✶, ✶✶✶).

	Region	Chick Wnt7a	Mouse Wnt7a	Similarity/ Difference
**CNS**	Telencephalon	✓	✓	=
	Diencephalon	✓ dorsal	✓ ventral and lateral	✶✶
	Midbrain	✓ lateral and ventral	✓ dorsal and lateral	✶✶
	Rhombencephalon	✓	✓	=
	Neural tube	✓ ventral midline	✓ ventral, more extensive	✶
**Facial**	Branchial arch	✓ posterior, restricted	✓ throughout	✶
	Frontonasal process	X	✓	✶✶✶
**Limb**	Mesenchyme	✓	✓	=
	Ectoderm	✓ dorsal	✓ HH20 dorsal; HH23+ proximal ventral	✶✶
**Other**	Eye	✓	X	✶✶✶
	Otic vesicle	✓ throughout	✓ throughout, highest levels dorsally	✶
	Heart	X	✓	✶✶✶
	Foregut	X	✓	✶✶✶
	Hindgut	X	✓	✶✶✶
	Somites	✓	X	✶✶✶

Supplementary Data Table 22: Comparison of Wnt7b Orthologue Expression in the Chick and Mouse at stages HH20-HH26 and TS15-TS19 respectively (Similar =, Degree of difference ✶, ✶✶, ✶✶✶).

	Region	Chick Wnt7b	Mouse Wnt7b	Similarity /Different
**CNS**	Telencephalon	✓	✓	=
	Diencephalon	✓ ventral and dorsal	✓ ventral and lateral, more complex	✶✶
	Midbrain	✓ dorsolateral and ventral	✓ dorsal and ventral, more complex	✶✶
	MHB	✓	X	✶✶✶
	Rhombencephalon	✓ ventral	✓ diffuse anteriorly, defined posteriorly	✶
	Neural tube	✓ ventral	✓ ventral, restricted	✶
**Facial**	Branchial arch	✓ distal	✓ ectoderm	✶
	Frontonasal process	✓	✓	=
**Limb**	Ectoderm	✓	✓	=
**Other**	Eye	✓ optic cup, cornea and lens	✓ ventral edge of optic cup, two patches of expression	✶✶
	Otic vesicle	✓ throughout	✓ throughout, highest levels dorsally	✶
	Lung	✓ mesenchyme	✓ mesenchyme; epithelium	✶✶
	Midgut	✓	X	✶✶✶
	Hindgut	X	✓	✶✶✶
	Body wall	X	✓	✶✶✶

Supplementary Data Table 23: Comparison of Wnt8 Paralogue (Wnt8a,Wnt8b) Expression in the Chick across HH20-HH26 (Similar =, Degree of difference ✶, ✶✶, ✶✶✶).

**Chick**	**Region**	**Wnt8a**	**Wnt8b**	**Similarities/ Differences**
**CNS**	Telencephalon	✓	✓	=
	Diencephalon	✓ dorsal	✓ either side of dorsal midline (stripes)	✶
	Midbrain	✓ dorsal and ventral	dorsal	✶✶
	Rhombocephalon	X	✓	✶✶✶
**Facial**	Branchial arch	✓ diffuse throughout all, highest in branchial arch 1	✓ diffuse throughout all branchial arches	✶
	Frontonasal Process	X	✓	✶✶✶
**Other**	Tail	✓	X	✶✶✶

Supplementary Data Table 24: Comparison of Wnt8 Paralogue (Wnt8a,Wnt8b) Expression in the Mouse across TS15-TS19 (Similar =, Degree of difference ✶, ✶✶, ✶✶✶).

Mouse	System	Wnt8a	Wnt8b	Similarities/ Differences
**CNS**	Telencephalon	✓	✓	=
	Diencephalon	✓ ventral; localised patch on dorsal anterior	✓ ventral anterior; either side of midline dorsally	✶✶
	Midbrain	✓	X	✶✶✶
	MHB	✓	✓	=
	Rhombencephalon	X	✓	✶✶✶
**Facial**	Branchial arches	✓	X	✶✶✶
	Frontonasal process	✓	X	✶✶✶
**Limb**	Mesenchyme	✓	X	✶✶✶
	Ectoderm	✓	X	✶✶✶
**Other**	Eye	✓ lens vesicle; optic cup	✓ optic cup	✶
	Otic vesicle	✓ throughout	✓ throughout, mesenchyme dorsally	✶✶
	Heart	✓ atria	✓ diffuse	✶✶
	Midgut	X	✓	✶✶✶
	Hindgut	✓	X	✶✶✶
	Body wall	✓ ectoderm	✓ anterior lateral line (from otic vesicle to forelimb) and lateral body wall	✶
	Tail	✓	X	✶✶✶

Supplementary Data Table 25: Comparison of Wnt8a Orthologue Expression in the Chick and Mouse at stages TS15-TS19 and HH20-26 respectively (Similar =, Degree of difference ✶, ✶✶, ✶✶✶).

	System	Chick Wnt8a	Mouse Wnt8a	Similarities/ Differences
CNS	Telencephalon	✓ dorsal anteriorly	✓ dorsal posterior telencephalic vesicles tapering mesially	✶
	Diencephalon	✓ dorsal	✓ ventral, localised patch dorsal anterior	✶✶
	Midbrain	✓ ventral and dorsal	✓ ventral	✶✶
	MHB	X	✓	✶✶✶
Facial	Branchial arches	✓ diffuse throughout all, highest in branchial arch 1	✓ diffuse	✶
	Frontonasal processes	X	✓	✶✶✶
Limb	Mesenchyme	X	✓	✶✶✶
Other	Eye	X	✓	✶✶✶
	Otic vesicle	X	✓	✶✶✶
	Heart	X	✓	✶✶✶
	Hindgut	X	✓	✶✶✶
	Body wall	X	✓	✶✶✶
	Tail	✓	✓	=

Supplementary Data Table 26: Comparison of Wnt8b Orthologue Expression in the Chick and Mouse at stages TS15-TS19 and HH20-26 respectively (Similar =, Degree of difference ✶, ✶✶, ✶✶✶).

	System	Chick Wnt8b	Mouse Wnt8b	Similarities/ Differences
**CNS**	Telencephalon	✓ anteriorly	✓ posterior and mesial	✶
	Diencephalon	✓ either side of dorsal midline (stripes)	✓ ventral, anterior; either side of midline dorsally	✶✶
	Midbrain	✓	X	✶✶✶
	MHB	X	✓	✶✶✶
	Rhombencephalon	✓	✓	=
**Facial**	Branchial arches	✓	X	✶✶✶
**Other**	Eye	X	✓	✶✶✶
	Otic vesicle	X	✓	✶✶✶
	Heart	X	✓	✶✶✶
	Midgut	X	✓	✶✶✶
	Body wall	X	✓	✶✶✶
